# Postmortem investigations and identification of multiple causes of child deaths: An analysis of findings from the Child Health and Mortality Prevention Surveillance (CHAMPS) network

**DOI:** 10.1371/journal.pmed.1003814

**Published:** 2021-09-30

**Authors:** Robert F. Breiman, Dianna M. Blau, Portia Mutevedzi, Victor Akelo, Inacio Mandomando, Ikechukwu U. Ogbuanu, Samba O. Sow, Lola Madrid, Shams El Arifeen, Mischka Garel, Nana Bukiwe Thwala, Dickens Onyango, Antonio Sitoe, Ima-Abasi Bassey, Adama Mamby Keita, Addisu Alemu, Muntasir Alam, Sana Mahtab, Dickson Gethi, Rosauro Varo, Julius Ojulong, Solomon Samura, Ashka Mehta, Alexander M. Ibrahim, Afruna Rahman, Pio Vitorino, Vicky L. Baillie, Janet Agaya, Milagritos D. Tapia, Nega Assefa, Atique Iqbal Chowdhury, J. Anthony G. Scott, Emily S. Gurley, Karen L. Kotloff, Amara Jambai, Quique Bassat, Beth A. Tippett-Barr, Shabir A. Madhi, Cynthia G. Whitney, the CHAMPS Consortium

**Affiliations:** 1 Department of Global Health, Rollins School of Public Health, Emory University, Atlanta, Georgia, United States of America; 2 Emory Global Health Institute, Emory University, Atlanta, Georgia, United States of America; 3 Center for Global Health, Centers for Disease Control and Prevention, Atlanta, Georgia, United States of America; 4 South African Medical Research Council Vaccines and Infectious Diseases Analytics Research Unit, University of the Witwatersrand, Johannesburg, South Africa; 5 Department of Science and Innovation/National Research Foundation: Vaccine Preventable Diseases, University of the Witwatersrand Faculty of Health Sciences, Johannesburg, South Africa; 6 US Centers for Disease Control and Prevention-Kenya, Kisumu, Kenya; 7 Centro de Investigação em Saúde de Manhiça [CISM], Manhica, Mozambique; 8 Instituto Nacional de Saúde [INS], Manhiça, Mozambique; 9 Crown Agents, Freetown, Sierra Leone; 10 Centre pour le Développement des Vaccins (CVD-Mali), Ministère de la Santé, Bamako, Mali; 11 Center for Vaccine Development and Global Health, University of Maryland School of Medicine, Baltimore, Maryland, United States of America; 12 Department of Infectious Disease Epidemiology, London School of Hygiene & Tropical Medicine, London, United Kingdom; 13 College of Health and Medical Sciences, Haramaya University, Harar, Ethiopia; 14 International Center for Diarrhoeal Diseases Research (icddr,b), Dhaka, Bangladesh; 15 Kisumu County, Kenya Department of Health, Kisumu, Kenya; 16 ICAP–Columbia University, Makeni, Sierra Leone; 17 Kenya Medical Research Institute (KEMRI) Center for Global Health Research, Kisumu, Kenya; 18 Universitat de Barcelona, Barcelona, Spain; 19 World Hope International, Makeni, Sierra Leone; 20 Department of Epidemiology, Johns Hopkins Bloomberg School of Public Health, Baltimore, Maryland, United States of America; 21 Division of Infectious Disease and Tropical Pediatrics, Department of Pediatrics, University of Maryland School of Medicine, Baltimore, Maryland, United States of America; 22 Ministry of Health and Sanitation, Freetown, Sierra Leone; 23 ISGlobal, Hospital Clinic, Universitat de Barcelona, Barcelona, Spain; 24 Catalan Institution for Research and Advanced Studies (ICREA), Barcelona, Spain; 25 Pediatric Infectious Diseases Unit, Pediatrics Department, Hospital de Sant Joan de Deu, University of Barcelona, Barcelona, Spain; 26 Consorcio de Investigacion Biomedica en Red de Epidemiologia y Salud Publica (CIBERESP), Madrid, Spain; University of Southampton, UNITED KINGDOM

## Abstract

**Background:**

The current burden of >5 million deaths yearly is the focus of the Sustainable Development Goal (SDG) to end preventable deaths of newborns and children under 5 years old by 2030. To accelerate progression toward this goal, data are needed that accurately quantify the leading causes of death, so that interventions can target the common causes. By adding postmortem pathology and microbiology studies to other available data, the Child Health and Mortality Prevention Surveillance (CHAMPS) network provides comprehensive evaluations of conditions leading to death, in contrast to standard methods that rely on data from medical records and verbal autopsy and report only a single underlying condition. We analyzed CHAMPS data to characterize the value of considering multiple causes of death.

**Methods and findings:**

We examined deaths identified from December 2016 through November 2020 from 7 CHAMPS sites (in Bangladesh, Ethiopia, Kenya, Mali, Mozambique, Sierra Leone, and South Africa), including 741 neonatal, 278 infant, and 241 child <5 years deaths for which results from Determination of Cause of Death (DeCoDe) panels were complete. DeCoDe panelists included all conditions in the causal chain according to the ICD-10 guidelines and assessed if prevention or effective management of the condition would have prevented the death. We analyzed the distribution of all conditions listed as causal, including underlying, antecedent, and immediate causes of death. Among 1,232 deaths with an underlying condition determined, we found a range of 0 to 6 (mean 1.5, IQR 0 to 2) additional conditions in the causal chain leading to death. While pathology provides very helpful clues, we cannot always be certain that conditions identified led to death or occurred in an agonal stage of death. For neonates, preterm birth complications (most commonly respiratory distress syndrome) were the most common underlying condition (*n* = 282, 38%); among those with preterm birth complications, 256 (91%) had additional conditions in causal chains, including 184 (65%) with a different preterm birth complication, 128 (45%) with neonatal sepsis, 69 (24%) with lower respiratory infection (LRI), 60 (21%) with meningitis, and 25 (9%) with perinatal asphyxia/hypoxia. Of the 278 infant deaths, 212 (79%) had ≥1 additional cause of death (CoD) beyond the underlying cause. The 2 most common underlying conditions in infants were malnutrition and congenital birth defects; LRI and sepsis were the most common additional conditions in causal chains, each accounting for approximately half of deaths with either underlying condition. Of the 241 child deaths, 178 (75%) had ≥1 additional condition. Among 46 child deaths with malnutrition as the underlying condition, all had ≥1 other condition in the causal chain, most commonly sepsis, followed by LRI, malaria, and diarrheal disease. Including all positions in the causal chain for neonatal deaths resulted in 19-fold and 11-fold increases in attributable roles for meningitis and LRI, respectively. For infant deaths, the proportion caused by meningitis and sepsis increased by 16-fold and 11-fold, respectively; for child deaths, sepsis and LRI are increased 12-fold and 10-fold, respectively. While comprehensive CoD determinations were done for a substantial number of deaths, there is potential for bias regarding which deaths in surveillance areas underwent minimally invasive tissue sampling (MITS), potentially reducing representativeness of findings.

**Conclusions:**

Including conditions that appear anywhere in the causal chain, rather than considering underlying condition alone, markedly changed the proportion of deaths attributed to various diagnoses, especially LRI, sepsis, and meningitis. While CHAMPS methods cannot determine when 2 conditions cause death independently or may be synergistic, our findings suggest that considering the chain of events leading to death can better guide research and prevention priorities aimed at reducing child deaths.

## Abstract 2

In an analysis of data from the Child Health and Mortality Prevention Surveillance (CHAMPS) network, Robert Breiman, Dianna Blau, and colleagues investigate how considering all conditions in the causal chain leading to death informs the identification of deaths attributable to various diagnoses.

## Author summary

**Why was this study done?:**

- More than 5 million deaths occur annually in children <5 years of age globally, mostly in low-income settings in Africa and South Asia.
- There is reason to believe that a substantial proportion of these deaths are preventable if there were effective programs that target the most common causes of death.

**What did the researchers do and find?:**

- We studied the causes of death in 7 sites in Africa and South Asia where child mortality is high by conducting surveillance for mortality and then collecting samples of tissue and other diagnostic specimens to perform pathology and diagnostic tests.
- In each country, a panel of clinical, laboratory, and epidemiological experts determined underlying, intermediate, and immediate causes of death for each child by studying the clinical data available before death, postmortem questionnaire surveys done with parents of the deceased, and postmortem pathology and testing.

**What do these findings mean?:**

- Up to now, most programs focus on a single cause of death (CoD) for each child referred to as the underlying condition.
- However, the findings suggest that most deaths have multiple conditions that contribute to death.
- Considering all of the conditions, rather than underlying condition alone, markedly changed the proportion of deaths attributed to various diagnoses, especially lower respiratory infection (LRI), sepsis, and meningitis.
- The findings suggest that considering the full chain of events leading to death can better guide priorities for research and strategies for mortality prevention.

## Introduction

An estimated 5.2 million children <5 years of age die each year [1]. Massive disparities underpin that figure: >4.3 million (83%) of these deaths occur in sub-Saharan Africa and South Asia [1]. As large as these numbers are, they reflect a drop of an estimated 7 million child deaths since 1990 [2]. Much of the decline in mortality has occurred in infants and children 1 to 5 years; consequently, neonatal deaths represent an increasing proportion of the mortality burden [3,4], estimated at 46% in 2019 [1]. The remaining childhood mortality burden and the associated geographic and economic status–based disparities are the focus of Sustainable Development Goal (SDG) 2030 objectives to “end preventable deaths of newborns and children under 5” and to reduce child mortality to less than 25 deaths per 1,000 live births in each country globally. To achieve these ambitious goals, data are needed that accurately quantify the leading causes of death, so that interventions targeting the common causes of death can be developed and implemented, especially in geographic locations that continue to experience high child mortality.

Results from in-depth investigations of the conditions contributing to child deaths may require new or modified modeling approaches to interpret and integrate the data within existing regional and global frameworks for calculating disease burden, including cause-specific mortality burden, which are often relied upon to prioritize investments in interventions to reduce mortality. Most burden of disease models work on a principle of one cause per death, to ensure that the “sum of cause-specific mortality rates was identical to total age-specific mortality rates,” as was stated in an early global disease burden report [5]. This approach became convention so that the causes of death reported do not exceed the number of deaths and because the sources of death information that enter into burden models, especially from low-income countries, have traditionally been vital registration, sample registration, and verbal autopsy data, sources that typically provide only a single underlying condition as the cause of reported deaths [1,5]. Among the methodologies used to ascertain cause of death (CoD), those derived from the verbal autopsy lack specificity with regard to most biological causes of death.

During the past decade, increasing use of postmortem specimens collected through minimally invasive tissue sampling (MITS—formerly referred to as minimally invasive autopsy) are illuminating the sequence of events leading to deaths in children [6–8]. Studies have illustrated high resolution in elucidating CoD with greater accuracy than can be expected from verbal autopsies [9] when postmortem pathology, microbiology, molecular, and other diagnostic testing are employed, including identifying specific pathogens responsible for infectious disease–associated deaths [10–12].

The Child Health and Mortality Prevention Surveillance (CHAMPS) network conducts standardized mortality surveillance following standardized protocols in high child mortality sites in 7 countries in Africa and South Asia [13]. This work includes detecting stillbirths and deaths in children under 5 years old, obtaining family consent for postmortem studies, collecting MITS specimens that undergo pathologic and diagnostic testing, and gathering verbal autopsy and clinical data to fully describe events leading to death. The full set of data is reviewed by a local panel to identify relevant conditions that led to each death, following ICD-10 and ICD-10 PM procedures, in a standardized process referred to as Determination of Cause of Death (DeCoDe) [14]. Evaluating all of the data collected, DeCoDe panels provide the underlying CoD, and if present, a separate immediate CoD and causes of death that are referred to as “antecedent or intermediate,” which are listed as ICD-10 codes between immediate and underlying. Each CoD report therefore provides a “causal chain,” shedding light onto the complete pathway from the first event initiating the cascade of occurrences that lead to the fatal outcome.

In contrast, mortality statistics have traditionally relied only on the underlying causes, completely neglecting other components in the chain of events leading to death. DeCoDe panels follow a fundamental counterfactual precept: For a cause to be listed in the causal chain, the panel members must conclude that if that condition was prevented (regardless of whether an effective prevention strategy currently exists) or effectively managed, the death would have been prevented. Findings from the initial 2 years of data collection from the first 5 CHAMPS sites to implement surveillance and postmortem studies demonstrated that most neonatal, infant, and childhood deaths had more than 1 condition in the causal chain, many of which were preventable [15].

We reason that focusing on only the underlying condition misses critical factors, leading to the deaths of children, thus, limiting the scope and potential of mortality prevention approaches, including developing new prevention and treatment modalities, diagnostics, improved clinical management, and specific strengthening of health systems. In this manuscript, we aimed to characterize how identifying, quantifying, and characterizing all conditions in the causal chain increases the potential for lives saved from interventions targeting leading causes of death when compared to considering only the underlying condition. In short, we posit that including multiple causes of death in burden models will present multiple ways to prevent death.

## Methods

We analyzed CHAMPS CoD data from all 7 sites to characterize the distribution of conditions that are listed as causes of death for neonates, infants (<28 days old), and children (12 months to <60 months). Surveillance methods and inclusion criteria for CHAMPS enrollment for MITS [13] have been described previously. Briefly, children who were <5 years of age at the time of death and were residents of a catchment area were eligible for enrollment. Parents or guardians were approached for consent for verbal autopsy and clinical chart abstraction; permission was also sought for specimen collection and testing for children whose deaths were identified within 24 hours of occurrence. The protocol was prospectively developed.

Site selection and characteristics have also been previously described [13]. Briefly, the CHAMPS network consists of 7 sites in sub-Saharan Africa and South Asia, each with a geographically defined catchment area: Baliakandi and Faridpur, Bangladesh; Bamako (Djikoroni Para and Banconi), Mali; Kersa and Harar, Ethiopia; Makeni (Bombali Shebora and Bombali Siari Chiefdoms), Sierra Leone; Manhiça, Mozambique; Siaya (Karemo) and Kisumu (Manyatta), Kenya; and Soweto and Thembelihle, South Africa. Sites were selected based on a variety of factors, including demonstrated mortality of greater than 50 deaths per 1,000 live births in children less than 5 years old at the time of site selection (2015), willingness of the local lead investigator to use a common, multisite protocol and to share data globally in real time, as well as ecologic and geographic diversity: We set out to have a distribution of rural, peri-urban, and urban (including informal settlement) sites, prevalence of HIV and malaria, and dispersed locations (geographically, to the degree possible) in sub-Saharan Africa. An additional key consideration was the possibility for a strong relationship between the site and the local ministry of health and/or national public health institute, to ensure that data collected contribute to national public health policy and actions. In addition, limited history of studies to understand disease burden were important components for selecting 2 sites (in Sierra Leone and Ethiopia).

Specimen collection and laboratory and pathology testing have also been described [15–18]. The study protocol is available at https://champshealth.org/resources/. Briefly, following written parental/guardian consent, trained CHAMPS technicians collected tissue specimens via standardized needle biopsies from lungs, heart, brain, liver, and bone marrow. Technicians also collected blood, cerebrospinal fluid (CSF), and nasopharyngeal and rectal swabs. Anthropometric data and photographs are also collected during the procedure to evaluate nutritional status and birth defects, respectively. Tissue specimens were reviewed by local and Centers for Disease Control and Prevention (CDC) pathologists using routine histopathology and special stains and immunohistochemistry [18]. Lung tissue, blood, CSF, and rectal and nasopharyngeal swabs were tested for 116 infectious pathogens using multiplexed TaqMan Array Cards [18], and blood and CSF were cultured using standardized microbiological methods. Data were abstracted from available clinical records (both child and maternal when applicable), and trained interviewers used appropriate language translations of the 2016 WHO verbal autopsy instrument to interview caregivers of enrolled deceased children [13,19]. All data for each deceased child from which MITS was collected were reviewed by DeCoDe panels [12] in each country. These local panels consist of clinicians (pediatricians and obstetricians), laboratorians, and public health specialists and determine the causes of death, assigning the underlying, antecedent (intermediate) and immediate causes of death with the appropriate ICD-10 codes following WHO ICD-10 and WHO application of ICD-10 to deaths during the perinatal period (ICD-PM) [19–21]. The ICD-10 codes are then grouped into broad categories based on the Global Burden of Disease groupings [22].

**Fig 1:**
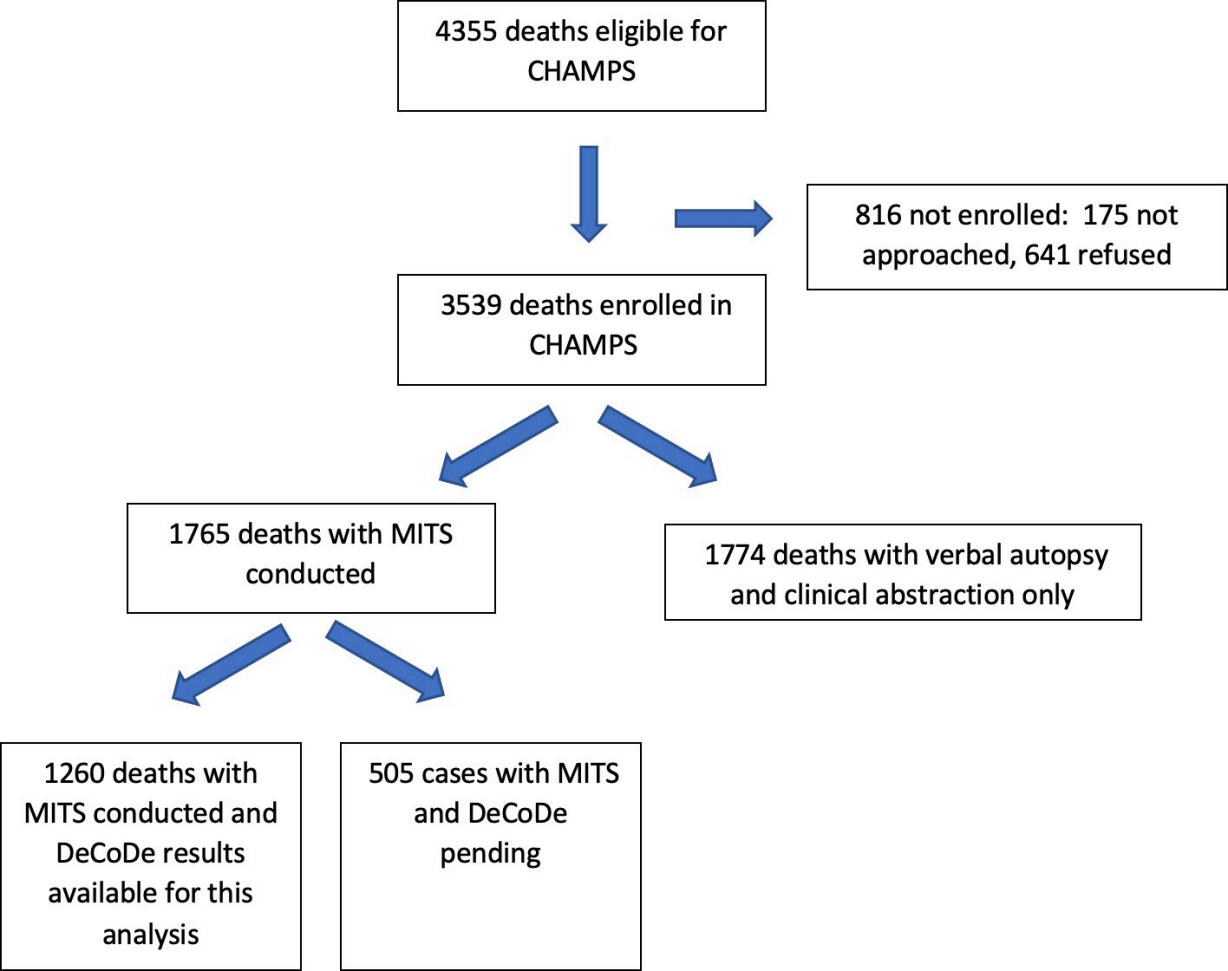
Flowchart of enrollment of deaths among children 0 to less than 5 years of age in CHAMPS sites, deaths with MITS procedure, and cases with CoDs determined by review panels (DeCoDe). Among 4,355 deaths eligible at the 7 CHAMPS sites, MITS was done for 1,765, and DeCoDe results were available for these analyses for 1,260 deaths. CHAMPS, Child Health and Mortality Prevention Surveillance; CoD, cause of death; DeCoDe, Determination of Cause of Death; MITS, minimally invasive tissue sampling.

A total of 4,355 deaths (excluding stillbirths) were identified with 3,539 enrolled across CHAMPS sites from 5 December 2016 through 30 November 2020, including 2,823 that were eligible for MITS (i.e., deaths identified within 24 hours of death in which the body was available and parents consented for the procedure) (Fig 1). Of these, MITS was done in 1,765 deaths and results from DeCoDe proceedings were available for 1,260 deaths, which represented the dataset used for this analysis. Stillbirths were excluded since the causes of death typically reflect maternal conditions.

We stratified analyses by deaths occurring during the first 27 days of life (neonates), from 28 days to <12 months of age (infants), and from 12 months to <60 months old (child). We defined the causal chain of mortality for each death to include any condition that appears as the underlying cause or was among antecedent or immediate cause categories, which, together, can include multiple causes. We defined “preterm birth conditions” to include low birth weight, hyaline membrane disease, necrotizing enterocolitis, and pneumothorax following the Global Burden of Disease groupings described above. For neonates with more than 1 type of preterm birth complication, the DeCoDe panels categorized them as having a preterm birth condition as the underlying CoD along with an additional preterm birth condition as either an antecedent or immediate cause. Similarly, other groupings, such as neonatal sepsis and perinatal asphyxia/hypoxia, could be found multiple times in the causal chain for the same death if different etiologies (i.e., multipathogen sepsis) or conditions that fell within that group were identified for that case.

We evaluated proportions of deaths by age category associated with each of the conditions listed in the causal chain and evaluated the additional causes in the causal chain for each underlying condition. Finally, we assessed the “attribution gain” (shown as x-fold increases) for each CoD when a causal chain assessment was used for each death by aggregating deaths by age group and comparing with the number of deaths that would be attributed to each cause when relying on underlying condition alone. We used medians, counts, and percentages to summarize data and used χ^2^ tests as appropriate to describe significant differences among number of causes. All analyses were conducted in SAS v 9.4.

Parents or guardians of deceased children provided written informed consent prior to collection of data, specimens, or information on living people (i.e., mothers). Ethics committees overseeing investigators at each site and at Emory University approved overall and site-specific protocols (Emory Institutional Review Board [IRB] #: 00091706). CDC relied for review on the Emory University committee for the overall protocol and on appropriate site ethical review committees where CDC staff were directly engaged at the site. The protocol is available at https://champshealth.org/wp-content/uploads/2021/08/CHAMPS-Mortality-Surveillance-Protocol-v1.3.pdf (S1 Protocol). Data can be accessed at champshealth.org, and requests can be made at the site to access additional datasets. Datasets are publicly available at (CHAMPS, 2021, “CHAMPS De-identified Dataset”; https://dataverse.unc.edu/dataset.xhtml?persistentId=doi:10.15139/S3/PMAAWG). This study is reported as per the Strengthening the Reporting of Observational Studies in Epidemiology (STROBE) guideline (S1 Checklist).

## Results

Among 1,260 neonatal, infant, and child deaths, a CoD was determined in 1,232; 28 (2%) were considered undetermined after review of all data. Of these 1,232 deaths, the DeCoDe process identified 51 separate underlying disease diagnoses (Tables 1–3). The number of additional conditions in the causal chain for each death ranged from 0 to 6, with a median of 1 (mean of 1.5, IQR 0 to 2) additional condition. About 1 in 3 (31%, 223/726) deaths in the neonatal age group were attributed to only an underlying cause without any additional causes, compared to 21% (56/268) and 25% (60/238), respectively, deaths among infants and children. The number of conditions in the causal chain is greater for deaths occurring in a health facility [mean 2.57, 95% CI (2.48, 2.65)] when compared with community deaths [mean 1.9, 95% CI (1.76 to 2.12)] (*p* < 0.001), regardless of age (Table 4). Among the top 5 underlying conditions, 4 (preterm birth complications, perinatal asphyxia, congenital birth defects, and neonatal sepsis) predominantly affect neonatal outcomes; malnutrition was listed as the underlying condition for 84 deaths (7% of all deaths and 17% of non-neonatal deaths). Other common (>10%) underlying conditions affecting under-fives in at least 1 age category included HIV, lower respiratory infections (LRIs), malaria, diarrheal diseases, congenital infection, and injury.

**Table 1:** Underlying causes for 741 neonatal deaths.

Underlying cause	# of deaths	%
Neonatal preterm birth complications	282	38%
Perinatal asphyxia/hypoxia	192	26%
Neonatal sepsis	79	11%
Congenital birth defects	68	9%
Congenital infection	27	4%
Neonatal aspiration syndromes	16	2%
Other neonatal disorders	15	2%
LRIs	11	2%
Neonatal encephalopathy	9	1%
Meningitis	5	0.7%
Chorioamnionitis and membrane complications*	3	0.4%
Syphilis	2	0.3%
All others (with only 1 case each)	17	2%
Undetermined	15	2%
**Total**	**741**	

* Placenta was rarely obtained and examined; however, clinical records were considered when making the diagnosis of chorioamnionitis.

LRI, lower respiratory infection.

**Table 2:** Underlying causes for 278 infant deaths.

Underlying cause	# of deaths	%
Malnutrition	38	14%
Congenital birth defects	37	13%
LRIs	32	12%
Neonatal preterm birth complications	32	12%
HIV	30	11%
Diarrheal diseases	18	7%
Malaria	13	5%
Sepsis	11	4%
Injury	6	2%
Other respiratory disease	5	2%
Other infections	5	2%
Sudden infant death syndrome	4	1%
Other neurological disorders	4	1%
Other neonatal disorders	4	1%
Congenital infection	4	1%
Cancer	3	1%
Poisoning	2	0.7%
Meningitis/encephalitis	2	0.7%
Paralytic ileus and intestinal obstruction	2	0.7%
Liver disease	2	0.7%
Other skin and subcutaneous diseases	2	0.7%
All other causes (with 1 each)	12	4%
Undetermined	10	4%
**Total**	**278**	

LRI, lower respiratory infection.

**Table 3:** Underlying causes for 241 child deaths.

Underlying cause	# of deaths	%
Malnutrition	46	19%
Malaria	39	16%
HIV	32	13%
Injury	23	10%
Congenital birth defects	18	7%
Diarrheal diseases	15	6%
LRIs	10	4%
Other respiratory disease	8	3%
Sepsis	6	2%
Other infections	5	2%
Meningitis	3	1%
Sickle cell disorders	3	1%
Tuberculosis	3	1%
Measles	3	1%
Cancer	3	1%
Other endocrine, metabolic, blood, and immune disorders	3	1%
Poisoning	2	0.8%
Liver disease	2	0.8%
Epilepsy	2	0.8%
Other	12	5%
Undetermined	3	1%
**Total**	**241**	

LRI, lower respiratory infection.

**Table 4:** Mean number of conditions in the causal chain for each age group by whether the death occurred in the community or in a healthcare facility.

Age group	Community		Facility		Overall (for age group)	
	*N*	Mean (95% CI)	*N*	Mean (95% CI)	*N*	Mean (95% CI)
Neonates	44	1.7 (1.3, 2.1)	697	2.4 (2.3, 2.5)	741	2.4 (2.3, 2.5)
Infant	82	1.9 (1.6, 2.2)	196	2.9 (2.7, 3.1)	278	2.6 (2.4, 2.8)
Child	75	2.1 (1.8, 2.4)	166	2.7 (2.5, 2.9)	241	2.5 (2.4, 2.7)
TOTAL	201	1.9 (1.8, 2.1)	1,059	2.6 (2.5, 2.7)	1,260	2.5 (2.4, 2.5)

### Neonatal deaths

Among 741 neonatal deaths, DeCoDe panels identified 27 different underlying disease diagnoses (Table 1). The most common were preterm birth complications (282, 38%), perinatal asphyxia (192, 26%), neonatal sepsis (79, 11%), and congenital birth defects (68, 9%). Excluding 15 deaths for which the CoD was undetermined, 503 (69%) deaths had 1 or more CoD beyond the underlying cause (mean = 1.4, IQR 0 to 2). Preterm birth complications include low birth weight, respiratory distress syndrome, prematurity, and bronchopulmonary dysplasia; of those neonatal deaths with preterm birth complications listed anywhere in the causal chain, 67% (234/347) had respiratory distress syndrome.

Among 282 neonatal deaths with preterm birth complications as the underlying condition, 256 (91%) had additional conditions in the causal chain, including 184 (65%) with another type of preterm birth complication (Table 5). Nearly half (137, 49%) of neonatal deaths with preterm birth complications as the underlying cause had other conditions not related to preterm birth complications within the causal chain. Indeed, more than half of the deaths with preterm birth complications or congenital birth defects as underlying conditions had ≥3 other diagnoses in the causal chain (Table 5). The most common conditions listed as immediate or antecedent causes (other than preterm birth complications) included neonatal sepsis [128 (45%)], LRI [69 (24%)], meningitis [60 (21%)], and perinatal asphyxia/hypoxia [25 (9%)] (Table 6). When known, the most common maternal factors associated with deaths due to preterm birth complications included hypertension (58/273, 21%), multiple gestation (39/273, 14%), and placental separation/antepartum hemorrhage (26/273, 10%). For deaths due to perinatal asphyxia, the most common maternal factors included obstructed labor and other complications of labor and delivery and placental separation/antepartum hemorrhage.

**Table 5:** Number of conditions in causal chain: Neonates.

Number of other conditions in causal chain	Underlying cause						
	Preterm birth complications (*n* = 282)	Perinatal asphyxia/hypoxia (*n* = 192)	Neonatal sepsis (*n* = 79)	Congenital birth defects (*n* = 68)	Congenital infection (*n* = 27)	All other causes (*n* = 78)	Total (*n* = 726)
0	26 (9%)	103 (54%)	36 (46%)	20 (29%)	4 (15%)	34 (44%)	223 (31%)
1	82 (29%)	51 (27%)	16 (20%)	20 (29%)	9 (33%)	18 (23%)	196 (27%)
2	77 (27%)	28 (15%)	15 (19%)	12 (18%)	7 (26%)	14 (18%)	153 (21%)
3+	97 (34%)	10 (5%)	12 (15%)	16 (24%)	7 (26%)	12 (15%)	154 (21%)

**Table 6:** Most common additional conditions (antecedent and immediate CoD) in the causal chain for the 5 leading underlying causes of neonatal deaths in CHAMPS.*

Antecedent and immediate causes of death^†^	Underlying cause					
	Preterm birth complications (*n* = 282)	Perinatal asphyxia/hypoxia (*n* = 192)	Neonatal sepsis (*n* = 79)	Congenital birth defects (*n* = 68)	Congenital infection (*n* = 27)	All other causes (*n* = 78)
Preterm birth complications^¥^	184 (65%)	18 (9%)	17 (22%)	12 (18%)	8 (30%)	10 (13%)
Low birth weight	12 (4%)	9 (5%)	15 (19%)	5 (7%)	7 (26%)	15 (19%)
Respiratory distress syndrome	162 (57%)	11 (6%)	9 (11%)	3 (4%)	4 (15%)	18 (23%)
Pulmonary hemorrhage	10 (4%)	0	1 (1%)	1 (1%)	0	0
Primary atelectasis	8 (3%)	0	1 (1%)	4 (6%)	0	0
Perinatal asphyxia/hypoxia	25 (9%)	6 (3%)	11 (14%)	9 (13%)	6 (22%)	8 (10%)
LRIs	69 (24%)	10 (5%)	7 (9%)	17 (25%)	4 (15%)	6 (8%)
Meningitis/encephalitis	60 (21%)	3 (2%)	14 (18%)	6 (9%)	1 (4%)	5 (6%)

* Excluding cases that only had 1 cause (which would be listed as the underlying condition) in the causal chain.

^†^ These conditions appear anywhere else (other than as an underlying condition) in the causal chain (i.e., as immediate or antecedent); thus, a single case in a column with that underlying cause can be counted in multiple rows if there is more than 1 contributing condition within that category (like with preterm birth conditions, which have multiple contributing conditions) in the causal chain.

^¥^ For multiple cases, there was >1 preterm birth complication listed as antecedent or immediate.

CHAMPS, Child Health and Mortality Prevention Surveillance; CoD, cause of death; LRI, lower respiratory infection.

### Infant deaths

Among 278 infant deaths, 31 different underlying disease diagnoses were identified (Table 2). Five conditions accounted for >50% of the underlying diagnoses, including malnutrition (38, 14%), congenital birth defects (37, 13%), LRI (*n* = 32; 12%), preterm birth complications (32, 12%), and HIV (30, 11%). Excluding 11 deaths for which the causes of death was undetermined resulted in 268 deaths with at least 1 cause determined; of these, 212 (79%) infant deaths had at least 1 additional CoD beyond the underlying cause (mean = 1.6; IQR = 1 to 3).

Among the 38 infant deaths with malnutrition as the underlying condition, 37 (97%) had a least 1 other condition in the causal chain (Table 7); the number of conditions in the causal chain ranged from 1 to 4 (median = 2, mean = 1.7). The most common conditions listed as immediate or intermediate causes included sepsis (19, 50%) and LRI (19, 50%) (Table 8). Among the 37 infant deaths with congenital birth defects as the underlying condition, 36 (97%) had at least 1 other condition in the causal chain (Table 7); the most common conditions in the causal chains for these deaths included sepsis (20, 54%), LRI (18, 49%), and meningitis (5, 14%) (Table 8).

**Table 7:** Number of conditions in causal chain: Infants.

Number of other conditions in causal chain	Underlying cause						
	Malnutrition (*n* = 38)	Congenital birth defects (*n* = 37)	LRI (*n* = 32)	Preterm birth complications (*n* = 32)	HIV (*n* = 30)	All other causes (*n* = 99)	Total (*n* = 268)
**0**	1 (3%)	1 (3%)	10 (31%)	0	2 (7%)	42 (42%)	56 (21%)
**1**	17 (45%)	9 (24%)	17 (53%)	4 (13%)	9 (30%)	28 (28%)	84(31%)
**2**	13 (34%)	13 (35%)	4 (13%)	6 (19%)	7 (23%)	14 (14%)	57 (21%)
**3+**	7 (18%)	14 (38%)	1 (3%)	22 (69%)	12 (40%)	15 (15%)	71 (26%)

LRI, lower respiratory infection.

**Table 8:** Most common additional conditions (antecedent and immediate CoD) in the causal chain for the 5 leading underlying causes of infant deaths in CHAMPS.*

Antecedent and immediate causes of death^†^	Underlying cause					
	Malnutrition (*n* = 38)	Congenital birth defects (*n* = 37)	LRIs (*n* = 32)	Preterm birth complications (*n* = 32)	HIV (*n* = 30)	All other causes (*n* = 99)
LRIs	19 (50%)	18 (49%)	6 (19%)	19 (59%)	22 (73%)	30 (30%)
Sepsis	19 (50%)	20 (54%)	12 (38%)	24 (75%)	12 (40%)	25 (30%)
Meningitis/encephalitis	2 (5%)	5 (14%)	3 (9%)	7 (22%)	3 (10%)	13 (13%)
Other infections	3 (8%)	4 (11%)	1 (3%)	4 (13%)	8 (27%)	3 (3%)
Diarrheal diseases	6 (16%)	0 (0%)	2 (6%)	1 (3%)	3 (10%)	2 (2%)

* Excluding cases that only had 1 cause (which would be listed as the underlying condition) in the causal chain.

^†^ These conditions appear anywhere else (other than as an underlying condition) in the causal chain (i.e., as immediate or antecedent); thus, a single case in a column with that underlying cause can be counted within multiple rows if there is more than 1 contributing condition within that category (like if there are >1 specific causes of LRIs, such as pneumococcal and *Klebsiella* etiologies) in the causal chain.

CHAMPS, Child Health and Mortality Prevention Surveillance; CoD, cause of death; LRI, lower respiratory infection.

### Child deaths

For 241 child (age 1 to 5) deaths, 4 conditions accounted for >50% of the underlying causes of death: malnutrition (46, 19%), malaria (39, 16%), HIV (32, 13%), and injury (20, 10%) (Table 1). Excluding 3 cases for which the CoD was undetermined resulted in 238 child deaths with at least 1 CoD determined; of these, 178 (75%) deaths had at least 1 CoD beyond the underlying cause (mean = 1.5; IQR = 0 to 2).

**Fig 2:**
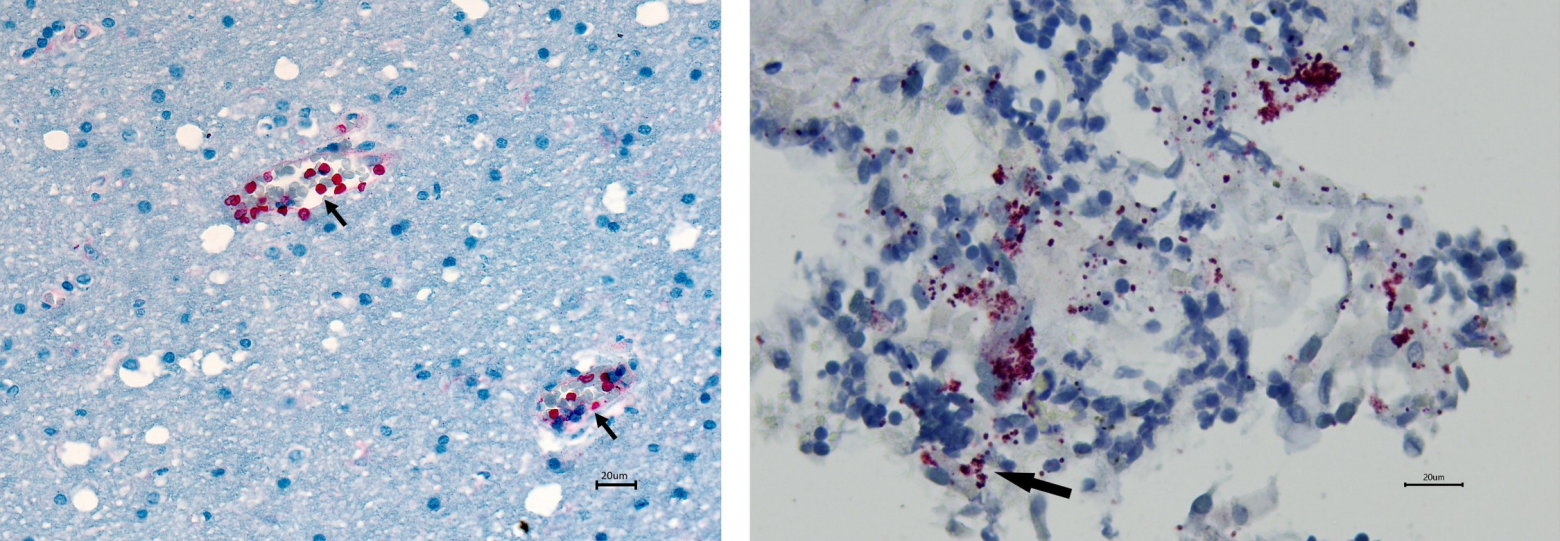
On the left, *Plasmodium falciparum* are visualized via staining by immunohistochemistry within red blood cells in small blood vessels (arrows) in the brain. On the right, clusters of *Streptococcus pneumoniae* bacteria are seen in the lung of the same child by immunohistochemistry (arrow pointing to one cluster).

Among the 46 child deaths with malnutrition as the underlying condition, all had at least 1 other condition in the causal chain (Table 9); the most common conditions listed as immediate or underlying causes included sepsis, LRI, malaria, and diarrheal disease (Table 10). While all sources of CHAMPS data contributed to identifying to multiple causes of death, pathologic findings, including immunohistochemistry, were often helpful for recognition and confirming them, as was the case for a child death due to both cerebral malaria and pneumococcal pneumonia (Fig 2).

**Table 9:** Number of conditions in causal chain for child deaths.

Number of other conditions in causal chain	Underlying cause						
	Malnutrition (*n* = 46)	Malaria (*n* = 39)	HIV (*n* = 32)	Injury (*n* = 21)	Congenital birth defects (*n* = 18)	All other causes (*n* = 80)	Total (*n* = 238)
0	0	22 (56%)	1 (3%)	6 (29%)	0	31 (39%)	60 (25%)
1	12 (26%)	7 (18%)	10 (31%)	7 (33%)	6 (33%)	23 (29%)	65 (27%)
2	14 (30%)	5 (13%)	15 (47%)	7 (33%)	5 (28%)	10 (13%)	56 (24%)
3+	20 (43%)	5 (13%)	6 (19%)	3 (14%)	7 (39%)	16 (20%)	57 (24%)

**Table 10:** Most common additional conditions (antecedent and immediate CoD) in the causal chain for the 5 leading underlying causes of child deaths in CHAMPS.*

Antecedent and immediate causes of death^†^	Underlying cause					
	Malnutrition (*n* = 46)	Malaria (*n* = 39)	HIV (*n* = 32)	Injury (*n* = 23)	Congenital birth defects (*n* = 18)	All other causes (*n* = 80)
LRIs	25 (54%)	4 (10%)	16 (50%)	7 (30%)	11 (61%)	24 (30%)
Sepsis	28 (61%)	4 (10%)	14 (44%)	11 (48%)	3 (17%)	18 (23%)
Malaria	13 (28%)	2 (5%)	7 (22%)	2 (9%)	3 (17%)	2 (3%)
Other respiratory disease	1 (2%)	7 (18%)	2 (6%)	1 (4%)	5 (28%)	7 (9%)
Diarrheal diseases	11 (24%)	1 (3%)	6 (19%)	0 (0%)	0 (0%)	3 (4%)

* Excluding cases that only had 1 cause (which would be listed as the underlying condition) in the causal chain.

^†^ These conditions appear anywhere else (other than as an underlying condition) in the causal chain (i.e., as immediate or antecedent); thus, a single case in a column with that underlying cause can be counted within multiple rows if there is more than 1 contributing condition within that category (in this for “all other causes,” there would be multiple specific conditions that appear as immediate or antecedent causes of death) in the causal chain.

CHAMPS, Child Health and Mortality Prevention Surveillance; CoD, cause of death; LRI, lower respiratory infection.

### Assessing the causal chain for mortality

Including conditions that appear anywhere in the causal chain (rather than underlying condition alone) markedly changed the proportion of deaths attributed for a variety of conditions, especially LRI, sepsis, and meningitis (Tables 11–13). For neonatal deaths, for example, including all positions in the causal chain resulted in 19-fold and 11-fold increases in attributable roles for meningitis and LRI, respectively (Table 11). For infant deaths, the proportion caused by meningitis and sepsis increased by 16-fold and 11-fold, respectively (Table 12); sepsis and LRI are increased 12-fold and 10-fold, respectively, for child deaths (Table 13).

**Table 11:** Increases in estimated contribution of leading causes of death when considering any location in the causal chain compared to only using the underlying CoD: Neonate (*n* = 726).

Condition	*n* (%) only as underlying condition	*n* (%) anywhere in causal chain	Fold increase
Meningitis	5 (0.7%)	94 (13%)	18.6
LRIs	11 (1.5%)	124 (17%)	11.3
Neonatal encephalopathy	9 (1.2%)	54 (7%)	5.8
Neonatal sepsis	79 (11%)	278 (37%)	3.4
Neonatal aspiration syndrome	16 (2%)	41 (5%)	2.5
Congenital infection	27 (4%)	59 (8%)	2.0
Perinatal asphyxia/hypoxia	192 (26%)	251 (34%)	1.3
Preterm birth complications	282 (38%)	347 (47%)	1.2
Congenital birth defects	68 (9%)	83 (11%)	1.2

CoD, cause of death; LRI, lower respiratory infection.

**Table 12:** Increases in estimated contribution of leading causes of death when considering any location in the causal chain compared to only using the underlying CoD: Infant (*n* = 268).

Condition	*n* (%) only as underlying condition	*n* (%) anywhere in causal chain	Fold increase
Meningitis	2 (1%)	35 (16%)	16.1
Sepsis	11 (5%)	119 (56%)	11.2
LRIs	32(15%)	140 (66%)	4.4
Diarrheal diseases	18 (8%)	31 (15%)	1.9
Malnutrition	38 (18%)	54 (25%)	1.4
Congenital birth defects	37 (17%)	40 (19%)	1.1

CoD, cause of death; LRI, lower respiratory infection.

**Table 13:** Increases in estimated contribution of leading causes of death when considering any location in the causal chain compared to only using the underlying CoD: Child (*n* = 238).

Condition	*n* (%) only as underlying condition	*n* (%) anywhere in causal chain	Fold increase
Sepsis	6 (3%)	84 (35%)	11.5
LRIs	10 (4%)	97 (40%)	10.0
Meningitis	3 (1%)	15 (6%)	3.5
Diarrheal diseases	15 (6%)	35 (14%)	2.3
Malnutrition	46 (19%)	66 (27%)	1.4
Malaria	39 (16%)	66 (27%)	1.7
Congenital birth defects	18 (7%)	21 (9%)	1.3

CoD, cause of death; LRI, lower respiratory infection.

## Discussion

Interventions targeting the common causes of neonatal, infant, and child deaths are needed to achieve SDG 2030 goals on reducing child mortality. A salient finding of our analyses of data from CHAMPS is the degree to which considering only the underlying CoD underestimates the role of several conditions in the mortality causal chain. The analyses showed that LRI, sepsis, and meningitis were the most underestimated conditions, depending on the age group; LRI was underestimated in all 3 age groups. Focusing on underlying conditions alone, as would be the case with most global burden of disease reports, 1.5%, 15%, and 4% of deaths in neonates, infants, and children, respectively, would be described as being due to LRI. However, if we consider LRI appearing anywhere within the causal chain, LRI would be associated with 17%, 66%, and 40% of deaths in neonates, infants, and children, respectively. That represents a range of 4.4- to 11.5-fold increases in the proportion of deaths in which the DeCoDe panel judged that preventing LRI would have averted death. In settings where resources to implement strategies to prevent or improve management are scarce, the findings of this work could help to clarify the potential value of reducing the impact of LRI on mortality, for example.

Sepsis and meningitis, both treatable and potentially preventable, represent 2 other frequently underestimated causes of death when only 1 cause is counted per death. We identified a >11-fold increase in sepsis-associated deaths when immediate and antecedent causes are considered for infants and children and a >3-fold increase for neonates. If underlying condition was only used, meningitis would be a massively underappreciated CoD in neonates and infants (>16-fold increase when immediate and antecedent causes are considered).

Previous work on mortality burden has focused on 1 cause per death and has not included postmortem pathological data, often relying on verbal autopsy, and limited antemortem clinical data [1,5]. The importance of recognizing all conditions in the causal chain is demonstrated when considering preterm birth complications. Preterm birth is well recognized as a cause of neonatal death [23,24]. Our findings suggest that a significant proportion of neonatal deaths in preterm babies had sepsis, LRI, and meningitis in the causal chain. In wealthy countries, well-managed preterm births survive the neonatal period and the babies often grow to lead normal, productive lives, highlighting the value of optimized clinical management in preterm babies. Improved diagnostic tools, therapeutics, and prevention strategies to reduce fatal sepsis, pneumonia, and meningitis in preterm babies have the potential of dramatically reducing mortality and improving outcomes. In addition, recognizing multiple maternal conditions that contribute to preterm births will help to focus on strategies for maternal care to improve birth outcomes. All of the above considerations are relevant for health system strengthening strategies, including infection control, antimicrobial drug stewardship, as well as support to critically ill infants, and research and development for better diagnostic tools, therapeutics, and vaccines [25]. An example of this potential is that CHAMPS has shown that sepsis (as well as LRI and meningitis) due to *Klebsiella pneumoniae* across CHAMPS sites and *Acinetobacter baumannii* (nosocomially transmitted predominantly in one site), often highly drug resistant, are important causes of neonatal and infant mortality [15]. This information has contributed to prioritizing diagnostic development for illness associated with these 2 pathogens, as well as evaluating *A*. *baumannii* monoclonal antibody therapeutics [26] and the potential for *K*. *pneumoniae* vaccines [25,27].

Further information on causes of LRI associated with mortality will also help with prioritizing new diagnostic and vaccine development, as well as basic lifesaving care (for example, use of oxygen). While ongoing data collection will provide more detail, early published data from CHAMPS have shown key contributions from bacterial pathogens like *K*. *pneumoniae*, *Streptococcus pneumoniae*, and *A*. *baumannii*, as well as among viral pathogens (multiple pathogens in causal chains for individual patients), especially respiratory syncytial virus, with varying contributions of each of these pathogens by age group and by whether the infections were acquired during hospitalization [15]. Like with syndromes and broad conditions (as shown in our current analyses), causal pathogens are found across the causal chain, often with >1 pathogen linked to mortality in a single case [15], further highlighting the importance of looking beyond underlying conditions when focusing on preventing mortality.

Age-specific analyses of child mortality are important for considering optimal immunization approaches and vaccine development; for instance, for causes of LRI that have impact during the neonatal period (like respiratory syncytial virus, RSV), maternal immunization (or use of monoclonal antibodies at birth) would be the approach to evaluate and optimize, given our current state of understanding of immune activation and responses. Information from the entire causal chain of mortality would be critical for consideration; if underlying diseases were the only focus and there was inability to identify causative pathogens, prioritization would be more subjective, relying on conjecture or extrapolations from data from clinical surveillance and testing of ill children.

The data most commonly used to prioritize targets for research and public health program strategies are global, regional, and local burden of disease reports [1,2,5]. They have provided foci for stakeholders to support programs to improve the health of children. Because existing reports include 1 death per child so that the total number of deaths reported equals 100% of deaths, the impact of conditions that contribute substantively to death when there are multiple causes is highly underestimated. Furthermore, CoD determinations, often based on hospital discharge data and verbal autopsy, can be associated with substantial misclassification. As a result, the utility of existing data for prioritizing mortality prevention interventions and research and development is dramatically reduced.

By using systematic collection and laboratory and pathologic evaluation of postmortem specimens, combined with review of available clinical data and verbal autopsies, the specificity of CoD reports increases [15], demonstrating that more than 1 condition usually contributes to death. Thus, focusing on 1 CoD severely limits the scope and potential impact of prevention programs and research and development into approaches that would target high-impact conditions.

We recognize a number of limitations in these analyses and the need for qualifications in our interpretations. Among 4,355 deaths that were detected within CHAMPS sites during the study period, MITS was done on 1,765. Nearly 35% (1,532/4,355) of deaths were detected after the time period allowed for MITS or burial had occurred; for deaths meeting criteria for MITS, parental consent was given in 63%. While the findings of this report represent a substantial number of deaths that have undergone comprehensive CoD determinations, there is a potential for bias for which deaths underwent MITS, which potentially reduces the representativeness of the findings. Likewise, while CHAMPS implemented a systematic site selection process with representativeness of high child mortality areas as an objective, given the variability of factors influencing child mortality, adjustments or ranges may need to be used in non-CHAMPS locations and in low- and middle-income countries with lower rates for child mortality (which may have fewer deaths with infectious diseases in causal chains).

We do not systematically collect social determinants of health (via “social autopsy”) [28] in all sites. Thus, DeCoDe panels do not include social and health system–associated contributors to death (like extreme poverty, poor obstetric care, availability of safe water and hygiene, and limited access to clinical care) in causal chains. Collecting such data in a standard form is under consideration for CHAMPS. We are currently not able to determine a causal framework for multiple causes of death, including whether 2 conditions cause death independently or whether they are synergistic with each other in leading to death. While the DeCoDe panels make a judgment that preventing or successfully managing each condition in the causal chain would have averted death, it is inevitable that at least some conditions occur after the child’s initial condition has worsened to a point that saving the life would be impossible even if those secondary conditions were treated optimally. As an example, a child near death from severe birth asphyxia might develop LRI, which accelerates the timing of death; ultimately, the immediate CoD might be determined to be LRI. Thus, the preventable burden will be somewhat overestimated for a proportion of conditions, when using the entire causal chain. CHAMPS is attempting to develop procedures to systematically determine conditions that are “agonal” versus truly causal, particularly challenging for deaths occurring outside of clinical settings.

Also, in some patients, sepsis, meningitis, and LRI in the causal chain reflect the same illness, during which a pathogen moves from the bloodstream to tissues or vice versa. In such cases of invasive infection, the same intervention (a pathogen-specific intervention, for example) would potentially prevent multiple conditions in the causal chain. However, 57% of such cases with >2 of the above syndromes had different pathogens responsible for the coexisting syndromes; 73% of children had sepsis, LRI, and/or meningitis caused by different pathogens, followed by 69% in infants and 38% in neonates.

In conclusion, the findings of this report provide insights on how public health action, clinical management, and research and priorities for the development of diagnostic, prevention, and treatment tools can be affected by considering the multiple conditions that cause death identified when comprehensive postmortem surveillance and investigation takes place. As data accumulate from CHAMPS sites, these insights will become more granular at the pathogen level, by region and by a variety of other factors including specifics on settings (such as hospital or community) for exposure prior to death. Emerging child mortality data from high mortality areas need to be continuously assessed and translated into lifesaving action to approach SDG 2030 child mortality goals.

## Supporting information

S1 Checklist STROBE guideline checklist. STROBE, Strengthening the Reporting of Observational Studies in Epidemiology.
(DOCX)

S1 Protocol The overall protocol for the CHAMPS mortality surveillance. CHAMPS, Child Health and Mortality Prevention Surveillance.
(DOCX)

## Abbreviations

CDC: Centers for Disease Control and Prevention
CHAMPS: Child Health and Mortality Prevention Surveillance
CoD: cause of death
CSF: cerebrospinal fluid
DeCoDe: Determination of Cause of Death
IRB: Institutional Review Board
LRI: lower respiratory infection
MITS: minimally invasive tissue sampling
RSV: respiratory syncytial virus
SDG: Sustainable Development Goal
STROBE: Strengthening the Reporting of Observational Studies in Epidemiology

## References

[1] United Nations Inter-agency Group for Child Mortality Estimation (UN IGME). Levels & Trends in Child Mortality. Report 2019. Estimates developed by the UN Inter-agency Group for Child Mortality Estimation. New York; 2019 [cited 11 Jan 2020]. Available from: https://childmortality.org/reports.

[2] You D, Hug L, Ejdemyr S, Idele P, Hogan D, Mathers C, et al. Global, regional, and national levels and trends in under-5 mortality between 1990 and 2015, with scenario-based projections to 2030: a systematic analysis by the UN Inter-agency Group for Child Mortality Estimation. Lancet. 2015;386 (10010):2275–86. doi: 10.1016/S0140-6736(15)00120-8 26361942

[3] UNICEF. Committing to child survival: a promised renewed. New York: Progress Report; 2015.

[4] McArthur JW, Rasmussen K, Yamey G. How many lives are at stake? Assessing 2030 sustainable development goal trajectories for maternal and child health. BMJ. 2018;360:k373. doi: 10.1136/bmj.k373 29449222 PMC5813301

[5] Murray CJL, Lopez AD. Mortality by cause for eight regions of the world: Global Burden of Disease Study. Lancet. 1997;349(9061):1269–76. doi: 10.1016/S0140-6736(96)07493-4 9142060

[6] Bassat Q, Ordi J, Vila J, Ismail MR, Carrilho C, Lacerda M, et al. Development of a post-mortem procedure to reduce the uncertainty regarding causes of death in developing countries. Lancet Glob Health. 2013;1(3):e125–e6. doi: 10.1016/S2214-109X(13)70037-8 25104253

[7] Ben-Sasi K, Chitty LS, Franck LS, Thayyil S, Judge-Kronis L, Taylor AM, et al. Acceptability of a minimally invasive perinatal/paediatric autopsy: healthcare professionals’ views and implications for practice. Prenat Diagn. 2013;33(4):307–12. doi: 10.1002/pd.4077 23457031

[8] Byass P. Minimally Invasive Autopsy: A New Paradigm for Understanding Global Health? PLoS Med. 2016;13(11):e1002173. doi: 10.1371/journal.pmed.1002173 27875535 PMC5119692

[9] Soleman N, Chandramohan D, Shibuya K. Verbal autopsy: current practices and challenges. Bull World Health Organ. 2006 Mar;84(3):239–45. doi: 10.2471/blt.05.027003 Epub 2006 Mar 22. . 16583084 PMC2627297

[10] Bassat Q, Castillo P, Martínez MJ, Jordao D, Lovane L, Hurtado JC, et al. Validity of a minimally invasive autopsy tool for cause of death determination in pediatric deaths in Mozambique: An observational study. PLoS Med. 2017 Jun 20;14(6):e1002317. doi: 10.1371/journal.pmed.1002317 eCollection 2017 Jun. . 28632739 PMC5478091

[11] Palhares AEM, Ferreira L, Freire M, Castillo P, Martínez MJ, Hurtado JC, et al. Performance of the minimally invasive autopsy tool for cause of death determination in adult deaths from the Brazilian Amazon: an observational study. Virchows Arch. 2019 Nov;475(5):649–658. doi: 10.1007/s00428-019-02602-z Epub 2019 Jun 14 31201504 PMC6861203

[12] Menendez C, Castillo P, Martínez MJ, et al. Validity of a minimally invasive autopsy for cause of death determination in stillborn babies and neonates in Mozambique: an observational study. PLoS Med. 2017 Jun 20;14(6):e1002318. doi: 10.1371/journal.pmed.1002318 eCollection 2017 Jun. . 28632735 PMC5478138

[13] Salzberg NT, Sivalogan K, Bassat Q, Taylor AW, Adedini S, El Arifeen S, et al. Mortality Surveillance Methods to Identify and Characterize Deaths in Child Health and Mortality Prevention Surveillance Network Sites. Clin Infect Dis. 2019;69:S262–S73. doi: 10.1093/cid/ciz599 31598664 PMC6785672

[14] Blau DM, Caneer JP, Philipsborn R, Madhi SA, Bassat Q, Varo R, et al. Overview and Development of the Child Health and Mortality Prevention Surveillance Determination of Cause of Death (DeCoDe) Process and DeCoDe Diagnosis Standards. Clin Infect Dis. 2019;69:S333–S41. doi: 10.1093/cid/ciz572 31598661 PMC6785670

[15] Taylor AW, Blau DM, Bassat Q, Onyango D, Kotloff KL, Arifeen SE, et al. Initial findings from a novel population-based child mortality surveillance approach: a descriptive study. Lancet Glob Health. 2020 Jul;8(7):e909–e919. doi: 10.1016/S2214-109X(20)30205-9 . 32562647 PMC7303945

[16] Rakislova N, Fernandes F, Lovane L, Jamisse L, Castillo P, Sanz A, et al. Standardization of minimally invasive tissue sampling specimen collection and pathology training for the Child Health and Mortality Prevention Surveillance Network. Clin Infect Dis. 2019;69(suppl 4):S302–10. doi: 10.1093/cid/ciz565 31598667 PMC6785668

[17] Diaz MH, Waller JL, Theodore MJ, Patel N, Wolff BJ, Benitez AJ, et al. Development and implementation of multiplex TaqMan array cards for specimen testing at Child Health and Mortality Prevention Surveillance site laboratories. Clin Infect Dis. 2019;69(suppl 4):S311–21. doi: 10.1093/cid/ciz571 31598666 PMC7108207

[18] Martines RB, Ritter JM, Gary J, Shieh WJ, Ordi J, Hale M, et al. Pathology and telepathology methods in the Child Health and Mortality Prevention Surveillance Network. Clin Infect Dis. 2019;69(suppl 4):S322–32. doi: 10.1093/cid/ciz579 31598668

[19] WHO. Verbal autopsy standards: the 2016 WHO verbal autopsy instrument. Geneva: World Health Organization; 2016.

[20] WHO. ICD-10: international statistical classification of diseases and related health problems, tenth revision. 2nd ed. Geneva: World Health Organization; 2004. 3376487

[21] WHO. The WHO application of ICD-10 to deaths during the perinatal period: ICD-PM. Geneva: World Health Organization; 2016.

[22] Global Burden of Disease Collaborative Network. Global Burden of Disease Study 2019 (GBD 2019) Cause List Mapped to ICD Codes. Seattle, United States of America: Institute for Health Metrics and Evaluation (IHME); 2020. doi: 10.6069/GHCW-8955

[23] UN Inter-agency Group for Child Mortality Estimation (IGME). Levels and trends in child mortality: report 2015. New York: UNICEF; 2015. Available from: http://www.childmortality.org

[24] Wolde HF, Gonete KA, Akalu TY, Baraki AG, Lakew AM. Factors affecting neonatal mortality in the general population: evidence from the 2016 Ethiopian Demographic and Health Survey (EDHS)—multilevel analysis. BMC Res Notes. 2019;12:610. doi: 10.1186/s13104-019-4668-3 31547855 PMC6757386

[25] Kollmann TR, Marchant A, Way SS. Vaccination strategies to enhance immunity in neonates. Science. 2020 May 8;368(6491):612–615. doi: 10.1126/science.aaz9447 . 32381718 PMC7734703

[26] Zurawski DV, McLendon MK. Monoclonal Antibodies as an Antibacterial Approach Against Bacterial Pathogens. Antibiotics (Basel). 2020 Apr 1;9(4):155. doi: 10.3390/antibiotics9040155 . 32244733 PMC7235762

[27] Feldman MF, Bridwell AEM, Scott NE, Vinogradov E, McKee SR, Chavez S, et al. A promising bioconjugate vaccine against hypervirulent Klebsiella pneumoniae. Proc Natl Acad Sci U S A. 2019;116:18655–63. doi: 10.1073/pnas.1907833116 31455739 PMC6744904

[28] Waiswa P, Kalter HD, Jakob R, Black RE, Social Autopsy Working Group. Bull World Health Organ. 2012 Jun 1;90(6):403–403A. doi: 10.2471/BLT.12.105718 . 22690025 PMC3370373

