# Supplementary material for: Postmortem investigations and identification of multiple causes of child deaths: An analysis of findings from the Child Health and Mortality Prevention Surveillance (CHAMPS) network
Source: PLoS Med. 2021 Sep 30;18(9):e1003814. doi: 10.1371/journal.pmed.1003814 (PMC8516282; doi:10.1371/journal.pmed.1003814)
Supplement: S1 Protocol — CHAMPS, Child Health and Mortality Prevention Surveillance. (DOCX) [file pmed.1003814.s002.docx]

**Child Health and Mortality Prevention Surveillance (CHAMPS) Network**

**Mortality Surveillance Protocol**

**Version 1.3 Date 19 October 2016**

[1. Introduction](#_h7y8voh41eel)

[1.1. Background](#_tyq39zuheskh)

[1.2 Limitations in ability to use current data for objectives](#_53hs1dgijy5v)

[1.3 Rationale for the CHAMPS Network Project](#_1kn1nm5mgkue)

[2. Study Objectives and Overview](#_lpl2wn2qxwt2)

[2.1 Primary Objectives](#_emxjxs51tc5c)

[2.2 Major Activities](#_hb8ipqlchcx)

[2.3 General Approach](#_6r0f1q2q48jy)

[3. Study Population](#_mq7vqwoipdok)

[3.1 Study Sites](#_c5512s9ajdmu)

[3.2 Mortality Surveillance](#_iareyyoq2a6s)

[Overview](#_a372vftuzk52)

[Facility-based Surveillance](#_y43bubu549k7)

[Community Based Surveillance](#_roaniot8uiwm)

[3.3 Inclusion Criteria](#_vy5r1r1cah0z)

[Mortality Surveillance](#_lyj63orx4apm)

[MITS and Clinical Data Collection](#_47joj3u2f4ko)

[3.4 Exclusion Criteria](#_l8xyomj3xrgd)

[Mortality Surveillance](#_s3v8cv74snkg)

[MITS and Clinical Data Collection](#_ipc76yqdeua)

[3.5 Sample Size Considerations](#_jcqafmndam29)

[Mortality Surveillance](#_z7i50txclg3h)

[MITS](#_rd79vkcjyliu)

[3.6 Representativeness of MITS Cases](#_7c3vdqt0d3d7)

[3.7 Recruitment](#_5665m63htd9v)

[4. Study Procedures/Methodologies](#_se23pz2qcepw)

[Mortality Surveillance](#_zg2uzy48o6t1)

[4.1 Minimally Invasive Tissue Sampling Procedure](#_1b90g9n9gw3g)

[Safety](#_nqqqnyk749aa)

[Figure 1. Examples of liver and brain tissue samples from a MITS procedure.](#_cotnxq7l7u3d)

[4.2 Clinical Data Collection](#_5yhqbql4dia4)

[4.3 Photography and Measurements](#_7vou6ahiwx9q)

[4.4 Verbal Autopsy](#_u0vbqaj2s1pn)

[4.5 Pregnancy and Neonatal Surveillance](#_m4b77mqybdsq)

[4.6 Demographic Surveillance](#_leun9rsvrr1r)

[4.7 CHAMPS Determining Cause of Death (DeCoDe) Panel Process](#_u73rkxbdl2v5)

[5. Training](#_vl8mnspe2q4y)

[5.1 Mortality Surveillance Procedures](#_2usfombo9kjp)

[5.2 MITS and Diagnostic Procedure Training](#_9140kb8s3lan)

[6. Storage and Archiving of Specimens](#_tzjlc3a9tu0u)

[6.1 Governance](#_s9edrp904sc5)

[6.2 Sample Access and Use](#_kyn1v9fhyon7)

[7 Data Management and Network Security](#_vbyvd2jmhy2d)

[7.1 Data Governance](#_otsfz4sgu6b1)

[7.3 Data Entry, Transmission, Security and Backup](#_tkxi7kctnjk5)

[7.4 Enrollment and Unique Identifier](#_yyd7s45qw4m1)

[7.5 Data Collection Instruments](#_88gi59olbu1g)

[7.6 Data Collection, Editing and Management](#_bp86p35hidk3)

[7.7 Quality Assurance and Quality Control Measures](#_sc1yr2cavf4q)

[8. Data analysis](#_yegahhh0ddlc)

[8.1 Data Analysis Plan](#_3n1e5xklj3g4)

[8.2 Limitations](#_u7ih0qlg8554)

[9. Managing Unexpected or Adverse Events](#_wop85m6ckua8)

[9.1 Response to new or Unexpected Findings and to Changes in the Study Environment](#_f9f6g1e4uz1g)

[9.2 Identifying, Managing and Reporting Adverse Events](#_z3zf937aac3x)

[10. Dissemination, Notification, and Reporting of Results](#_hlds4uspcczp)

[10.1 Notification of Participants of their Individual Results](#_j57ov90h6kd)

[10.2 Notification of Communities of Study Findings](#_8bqyhr1smpxz)

[10.3 Anticipated Products Resulting from the Project and Their Use](#_1p6fgrjjh0qb)

[10.4 Dissemination of Results](#_49fwoh2o3n14)

[11. Ethical and Human Subjects Considerations](#_6yq50z9rv6fe)

[11.1 Community Entry and Engagement Strategy](#_bhwloxmpmxrn)

[11.2 Informed Consent](#_hf789nc6ydas)

[11.3 Risks and Benefits](#_ftu92pi6v29t)

[11.3.1 Risks](#_bpcwxwvbd4yr)

[11.3.2 Benefits](#_iz6zddiylexn)

[11.3.3 Voluntariness, Privacy and Confidentiality](#_7asy5mucxdvz)

[11.3.4 Public Safety Considerations](#_yyqnr5vy6dmo)

[12. References](#_8v6aqvahtrwx)

[13. Appendices](#_czkvto7afx9)

[Appendix 1. Lab and Diagnostics Plan](#_bh8fdwkyly4d)

[Appendix 2: Data Access Plan](#_vwesnyglib8j)

[Appendix 23: Informed Consent Form](#_u80zwta9715t)

Abbreviations

| BMGF | Bill and Melinda Gates Foundation |
| --- | --- |
| CDA | Complete Diagnostic Autopsies |
| CDC | Centers for Disease Control and Prevention (Atlanta, Georgia, USA) |
| CHAMPS | Child Health and Mortality Prevention Surveillance Network |
| CPL | CHAMPS Pathology Laboratory |
| CSF | Cerebrospinal Fluid |
| DHS | Demographic and Health Surveys |
| DSS | Demographic Surveillance System |
| EGHI | Emory Global Health Institute |
| HDSS | Health and Demographic Surveillance System |
| IANPHI | International Association of Public Health Institutes |
| IRB | Institutional Review Board |
| IT | Information Technology |
| LMIC | Lower and Middle Income Country |
| MIA | Minimally Invasive Autopsy |
| MITS | Minimally Invasive Tissue Sampling |
| MoH | Ministry of Health |
| NGO | Non-Governmental Organization |
| NPHI | National Public Health Institute |
| PII | Personal Identifiable Information |
| PO | Program Office |
| SAE | Serious Adverse Event |
| SOP | Standard Operating Procedure |
| QA | Quality Assurance |
| QC | Quality Control |
| TAC | Taqman Array Card |
| VA | Verbal Autopsy |
| WHO | World Health Organization |
|  |  |

#

#

Definitions

- **Catchment area**: Area from which hospital draws patients or defined DSS area, as appropriate for site.
- **Child:** For the purposes of this protocol, we define a child as an individual under the age of 18 years. CHAMPS will focus initially on children under the age of 5 years (60 months).
- **Death**: Assumed irreversible cessation of all cardiorespiratory function, as certified by the attending clinician or confirmed by study staff.
- **Infant:** A child under the age of 12 months.
- **Neonatal:** Referring to an infant under the age of 28 days
- **Notified stillbirth**: A stillbirth reported through CHAMPS mortality surveillance in health facilities or community, pregnancy surveillance, or demographic surveillance
- **Notified under- 5 death**: All deaths occurring among children under 5 years of age that are reported through CHAMPS mortality surveillance in health facilities or communities, demographic surveillance, or pregnancy surveillance, or reported/notified through any other mechanism.
- **Stillbirth**: No spontaneous breathing or movement at time of delivery AND at least one of the following: 1) weighing 1000 grams or more or 2) estimated gestational age > 28 weeks
- **Under 5 years**: Postnatal age less than 5 years (60 months) at time of death.

#

Key Personnel and Participating Institutions

**Site investigators**

#

#

Abstract

Despite reductions over the past two decades, childhood mortality remains high, particularly in low income settings in sub-Saharan Africa and south Asia. In lower and middle income countries (LMIC), individuals often die without having been seen by qualified medical personnel and are frequently buried or cremated without a documented medical history, before an evaluation of the cause of death can be conducted; many times these deaths are not counted at all. As a result, overall mortality is underestimated, and because modeling is required, using a variety of assumptions and relying on non-specific data, calculations of the local, regional and global burden of specific causes of mortality result in dramatically conflicting numbers and substantial uncertainty. Credible and accurate mortality data would enable policy-makers, stakeholders, donors to effectively target the leading causes of childhood mortality for interventions, a critical component for achieving the Sustainable Development Goal for eliminating preventable childhood deaths.

The minimally invasive tissue sampling (MITS) procedure, formerly known as the minimally invasive autopsy (MIA), was developed as an approach to reduce uncertainty around cause of death determination in high mortality settings, where clinical and postmortem diagnoses are nearly always unavailable, and where performance of complete diagnostic autopsy would not be feasible. The procedure uses biopsy needles to obtain post-mortem samples of lung, brain, liver and other organs for histopathologic, microbiologic, and molecular examination to help determine cause of death.

The CHAMPS network will track the causes of under-5 mortality and stillbirths at sites in sub-Saharan Africa and South Asia through epidemiologic surveillance of under-5 deaths and stillbirths utilizing MITS, laboratory diagnostics including conventional and advanced histopathology and molecular screening of various pathogens, verbal autopsy, and available clinical and demographic data. Sites will establish notification systems in health facilities and within the community, which will report all under-5 deaths and stillbirths to the local CHAMPS team within 24 hours of the child’s death or delivery of stillbirth. The CHAMPS team will contact parents or guardians and request consent to perform MITS and laboratory investigation for the presence of infectious and other etiologies for the child’s death or stillbirth. Tissue samples will be analyzed by histopathology at both the site and central pathology laboratory, while bodily fluid samples will be analyzed primarily by the site laboratory. Target sample size will initially be 50-200 MITS cases per year for each site, progressively increasing as site resources allow. The site will also aim to perform standard verbal autopsies and collect any available clinical and demographic data on all notified child deaths and stillbirths. Though VA has limited specificity for some causes of death, VA data on deaths for which MITS cannot be obtained will increase the capacity to define the scope of childhood mortality in each CHAMPS site. Demographic and pregnancy surveillance capabilities of sites will be strengthened, as needed. Final cause of death determination for MITS cases will be made by an expert panel of clinicians, pathologists, epidemiologists, and laboratorians, which will review all available data on each case and assign a final cause of death. Cause of death for non-MITS cases will be determined by automated and/or physician review of the available VA and clinical data.

This document will serve as a model mortality surveillance protocol for all CHAMPS surveillance sites. Data will be collected in a uniform way, using standardized CHAMPS data collection tools and following standard operating procedures developed for all sites within the CHAMPS network, to allow aggregation into a de-identified combined dataset across the network. Each site will adapt this protocol with relevant minor local adjustments. Site-adapted protocols will be reviewed by the CHAMPS PO to ensure consistency in procedures and data collection across sites, and they will be subsequently approved by respective local Institutional Review Boards or ethics committees prior to implementation.

# 1. Introduction

## 1.1. Background

Despite reductions over the past two decades, childhood mortality remains high, particularly in low income settings in sub-Saharan Africa and south Asia. In lower and middle income countries (LMIC), individuals often die without having been seen by qualified medical personnel and are frequently buried or cremated without a documented medical history, before an evaluation of the cause of death can be conducted; many times these deaths are not counted at all. Even for those that die at a health facility, determining cause of death is often difficult due to the scarcity of diagnostic tools and multiple coexisting illnesses, which often leads to an incorrect or imprecise physician ascribed cause of death (Mushtag and Ritchie, 2005; Gupta et al., 2014). As a result, many estimates of the main causes of global and cause-specific mortality are based on modeling using data which primarily comes from verbal autopsies, a methodology which is the subject of increased scrutiny and debate (Boerma and Mathers, 2015; Murray et al, 2015). The resulting lack of solid empirical data leaves scientists, as well as stakeholders, without a good sense of what kills many people throughout the world. Uncertainty around the true causes of death in turn limits the effectiveness of national public health programs and hampers donors’ decision-making about the most beneficial use of their resources globally.

Complete diagnostic autopsies (CDA) are recognized as the most comprehensive and complete method to estimate cause of death (Fligner et al, 2011). However, due to cultural, financial, religious, and physical barriers, particularly in developing countries, CDAs are rarely undertaken in such resource-poor environments (Turner et al, 2012; Cox et al, 2011; Oluwasola et al, 2009). Additionally many deaths within lower middle income countries occur outside of healthcare facilities, precluding CDAs in an environment not developed to respond to such events. Those deaths that occur in the community may not be tracked or identified quickly enough for autopsy or postmortem examination to occur (Lishimpi et al, 2001; Ugiagbe & Osifo, 2012). This may be especially problematic for stillbirths and neonates who die in the community. This may introduce additional bias to global burden of disease estimates since the magnitude and causes of community-based deaths may be different than those that occur in the facility.

Instead of CDAs, the World Health Organization (WHO) recommends the use of verbal autopsy (VA) as a non-invasive alternative (Butler, 2010; Byass, 2014; Gareene, 2014; Jha, 2014). VA involves structured interviews with individuals close to the deceased, through which a cause of death is derived. However, VAs alone as a method to determine cause of death are hampered by the lack of objective diagnostic information, substantial recall bias, and they cannot distinguish between diseases with similar clinical presentations; thus, the specificities of VA for pathogen-specific causes of death are low for nearly all infectious disease etiologies. Despite these limitations, VA remains in many settings the best and most feasible standardized source of data regarding causes of death.

In health facility based settings, cause of death is generally derived from a clinician’s diagnosis of disease that led to death. However, research indicates that this methodology is often also inaccurate, with discordant diagnoses between autopsy findings and clinical diagnoses in close to half of cases (Roulson et al 2005). Therefore, with the imperfections inherent in VA methodology, the impracticality of CDAs in resource-poor settings, and the inaccuracy of clinician ascribed cause of death, an alternative is necessary in order to fill the knowledge gap on what is killing children in the LMICs.

**Minimally Invasive Tissue Sampling (MITS) Procedure**

The minimally invasive tissue sampling (MITS) procedure was developed to reduce the uncertainty regarding causes of death in developing countries (Bassat et al, 2013). MITS are potentially faster, much less disfiguring (thus, more acceptable), and less resource-intensive than CDAs (Vogel, 2012). MITS may provide the same basic data on many (especially infectious disease) causes of death and may be more feasible, thereby increasing the ability to conduct them on a population level (Ben-Sasi et al, 2013). MITS was first proposed in the literature in 1995 by Avrahami, et al (Avrahami et al, 1995). Multiple studies have explored the use of MITS to understand its clinical value and its potential for replacing conventional autopsy. While most studies have focused on developed countries as well as non-infectious causes of death, e.g. birth defects (Sebire et al, 2012; Fan et al, 2010; Breeze et al, 2011; Weustink et al, 2009), some recent projects in LMICs are working to validate the technique against the gold standard complete autopsy method in LMICs (Martinez et al 2016; Castillo et al 2015).

The MITS procedure involves extracting tissue specimens (and body fluids sampling) from a predefined set of organs without actually having to open the body, and undertaking histopathologic, microbiologic, and molecular investigations. Research thus far indicates that MITS should be conducted within 24 hours of a death (Castillo et al 2015, Martinez et al 2016), if the corpse is not refrigerated, and therefore, community death notification systems are vital in ensuring rapid response. It should be noted that autopsy information, whether obtained from full autopsy or MITS, should preferably be considered in conjunction with clinical findings (if available) and/or verbal autopsy information.

## 1.2 Limitations in ability to use current data for objectives

Today, the global health community lacks consistent, accurate, and timely infectious disease epidemiology and surveillance data to inform strategy and enable critical decisions for reducing childhood and perinatal mortality. Due to the limitations of the current cause of death methodologies, there is a pressing need for additional research to determine the best method to determine cause of death in LMICs, especially among children under five and stillbirths. A lack of quality primary data across key geographies has led to large gaps in knowledge and has necessitated a reliance on modeling. Additionally, data that are available are gathered through non-standardized processes into systems with limited ability to integrate across disease areas, funding streams, or bureaucracies, limiting stakeholders’ ability to integrate, analyze, compare, make inferences and take timely actions. Finally, the availability of primary data may often be delayed for years due to differing incentives among stakeholders, resulting in a lagging view of evolving epidemiology. This combination of factors restricts the ability of global stakeholders as well as national leaders to make evidence-based decisions such as prioritizing product development, appropriately targeting interventions, measuring the impact of interventions, and refining strategies to address changing epidemiology.

## 1.3 Rationale for the CHAMPS Network Project

In response to the limitations of currently available data, the Child Health and Mortality Prevention Surveillance (CHAMPS) Network was established with the aim to develop a long-term network of high-quality sites to collect robust and standardized longitudinal data with the overarching objective of understanding and tracking the preventable causes of childhood death globally. The resulting data will provide evidence needed to support the goal of significantly reducing child deaths in lower-resource countries. This network is led and managed by the CHAMPS Network ‘Program Office’ (PO) under the direction of the by Emory Global Health Institute ([EGHI](http://www.globalhealth.emory.edu/)), which will coordinate and oversee activities to the sites participating in the network and provide the necessary resources to implement the protocols and related activities in the sites. The project is funded through a grant from the Bill and Melinda Gates Foundation (BMGF).

The CHAMPS Network’s goal - to provide accurate, timely and reliable data for decision-making on the causes of death for children under age 5 and stillbirths - will provide the answers needed to support the goal of significantly reducing child deaths in lower-resource countries. Timely and accurate data (including a dashboard resource) generated by the initiative will inform the efforts of funding agencies, ministries of health, national public health institutes (NPHI), funding agencies, non-governmental organizations focused on reducing mortality, scientists, clinicians, government leaders, journalists and the public. The overall CHAMPS Network, when fully realized, has the goal of bringing to the world the knowledge needed to drive interventions to dramatically reduce early childhood death and disability, including a long list of potentially crucial contributing factors that may underlie the progression from severe illness to death.

Because of its primary emphasis on documenting cause of death, the CHAMPS Network’s objectives differ from those of typical surveillance programs and studies focusing on disease etiology. The scope is also broad, aiming to capture both perinatal causes of deaths (including stillbirths and neonatal mortality) and deaths in infants and children <5 years, and deaths caused by both infectious and noninfectious etiologies. Tissue and body fluid sampling methods will be coupled with advanced laboratory techniques, rarely applied in settings with high child mortality rates; these data will be used to attribute cause of death as accurately and as specifically as possible.

The CHAMPS Network data from each site will be crucial for optimally defining mortality burden, designing and implementing public health interventions, and measuring impact of new programs. Such results will in turn provide evidence for designing and scaling-up strategies to dramatically reduce child mortality in sub-Saharan Africa and South Asia.

# 2. Study Objectives and Overview

## 2.1 Primary Goal and Objectives

The primary CHAMPS Network goal is to track the causes of death for children under five years of age in surveillance sites in sub-Saharan Africa and South Asia by:

1. Targeting areas with high under-five mortality rates (>50 child deaths per 1000 live births),
2. Prioritizing preventable stillbirths, neonatal and under 5 deaths,
3. Utilizing minimally invasive tissue sampling (MITS) (also known as minimally invasive autopsy, or MIA) to determine the cause of death for many conditions and defining the conditions/types of deaths (like injuries, birth asphyxia, congenital malformations) for which other approaches would be required,
4. Including identification of specific pathogens and contributing risk factors associated with death, and
5. Including accurate measures of causes of stillbirth and neonatal mortality.

Specifically, the program will have the following primary objectives:

1. **Ascertain the cause(s) of death** for **notified** stillbirths and under-five deaths within 4 months of death as follows:
   1. Identify and **enumerate all under-5 deaths and stillbirths** in the catchment area
   2. Obtain **MITS** on as many under-5 deaths and stillbirths per year as possible (minimum: 50 cases in first year per surveillance site)
   3. Determine cause of death using all available, laboratory, histopathologic, clinical, and maternal and verbal autopsy data.
      1. Finalize cause of death determination on ≥80% of MITS cases within 4 months of death.
   4. Obtain **verbal autopsies** and available maternal and infant **clinical data** on all eligible notified under-5 deaths and stillbirths in the site’s catchment area to arrive at a cause of death for all notified cases without MITS data
2. Estimate the **total expected number of under-5 deaths and stillbirths in the catchment area** using denominators based on a variety of potential sources including: DSS enumeration, census data, pregnancy surveillance and/or results from health care utilization surveys as available
3. Estimate **overall and cause-specific mortality rates** (stillbirth and under-5) in the catchment area by combining the cause of death data for all notified cases with the denominators from demographic surveillance and other sources

## 2.2 Major Activities

The following major activities will be conducted to achieve the goals and aims of this protocol:

1. Initiate epidemiologic mortality surveillance in healthcare facilities and communities to enumerate deaths of children under 5 years (including stillbirths) in catchment area.
   1. Utilize minimally invasive tissue sampling (MITS) procedures on as many deaths as possible (initially at least 50 pediatric deaths [including stillbirths] per site in the first year, increasing the numbers progressively thereafter).
   2. Conduct verbal autopsy (VA) on all under-5 deaths and stillbirths.
   3. Assess and determine causes of death via systematic review of all available, relevant data for each death.
2. Perform in-process quality assurance (QA) and quality control (QC) measures, including monitoring and training to ensure high quality data and protocol compliance.
3. Create an archive of clinical specimens in each site country, as well as contribute to an international archive located in Atlanta (US) (see annex for more details on this).
4. Disseminate results to family members, community members, local and national public health authorities, and other key stakeholders on a routine basis.

## 2.3 General Approach

The CHAMPS Network will systematically identify and document causes of stillbirth and under-5 mortality. Surveillance will occur in hospitals, health centers, and communities within a defined population in each CHAMPS Network site. CHAMPS will gather data to determine cause of death for children who die in the catchment areas through the use of MITS procedures augmented with VA as well as maternal and child clinical and laboratory data. The data elements collected will allow for calculation of overall and cause-specific mortality for various conditions (e.g. diarrhea, respiratory illness, febrile illness, birth asphyxia) in the study population, levels of severity, delays in accessing care, and healthcare utilization data. Linkages with facility information will allow data collection for pathogen-specific mortality rates and pathogen-attributable fractions.

The final cause of death determination for MITS cases will be performed by a panel called the Determination of Cause of Death (DeCoDe) panel, which will review all available data for each case, including: 1) VA findings; 2) clinical information, including details about delivery and maternal obstetric history, if available; 3) microbiology, molecular biology (TaqMan Array Cards, TAC), HIV, TB, malaria findings; 4) surveillance site histopathology findings; 6) Central Pathology Lab histopathology results which could include special stains, immunohistochemistry, and PCR findings in selected cases .Specific and standardized operating procedures (SOPs) regarding the methods utilized by this panel to reach a final diagnosis will be prepared (see section 4.5).

Many stillbirths and under-5 deaths detected at the surveillance site will not undergo MITS or specimen collection due to resource constraints, timing, or parental refusal of MITS. Cause of death for non-MITS cases will be determined by automated and/or on-site expert review of all available VA and clinical data for each death.

While causes of death may fall into broad categories, we expect data from the CHAMPS Network to generate more accurate information than previous studies, which in turn will allow adjustments to models estimating global, cause-specific mortality. Data will be collected in a uniform way to allow aggregation into a de-identified combined dataset across the network.

This document will serve as a model protocol for all sites. Data will be collected in a uniform way, using standardized data collection tools and following standard operating procedures (SOP) developed for all of the sites within the CHAMPS network. Site-specific protocols will be reviewed by the CHAMPS PO to ensure consistency in procedures and data collection across sites, and they will be subsequently approved by respective local IRBs or ethics boards prior to implementation.

# 3. Study Population

## 3.1 Study Sites

Study sites are being selected in LMICs in South Asia and Sub-Saharan Africa with the principal goal of elucidating the etiology of mortality in children under five years old. Initially the CHAMPS Network will consist of 3-6 sites in sub-Saharan Africa and South Asia, each consisting of >100,000 total population or with at least 15,000 children <5 years of age. Sites have or will establish an enumerated population, either through a Health and Demographic Surveillance System (HDSS) or high-quality census/vital registration (defined as 100% estimated completeness and coverage of the CHAMPS catchment area). Each site is in a high under-5 mortality area, defined as U5MR >50 deaths per 1000 live births. Once the initial 6 sites become a functioning network, additional sites will be added during a second phase as soon as practical.

Sites will be closely linked to the PO through an established formal reporting structure and to each other through the establishment of a Community of Practice (including an annual meeting, on-site and virtual training, regional meetings, and other ongoing communication strategies). Sites will conduct CHAMPS Network activities according to CHAMPS SOPs in a uniform way to allow aggregation into a combined dataset across the network.

INSERT SITE SPECIFIC INFORMATION HERE

## 3.2 Mortality Surveillance

### Overview

All stillbirths, neonatal, and under-5 child deaths within the site’s catchment area will be enumerated to ensure completeness of death reporting and mortality rate calculations. Basic information will be collected about each death, such as dates of birth and death, contact information (so the family may be contacted for further consent and data collection), and brief circumstances of the child’s death (2-3 sentence narrative).

After CHAMPS surveillance activities have been initiated at a site, the enumeration of notified deaths will consist of triangulated data from: healthcare facility-based death notification (expected within 24 hours); community-based death notification (expected within 24 hours); pregnancy surveillance; and demographic surveillance.

Notified deaths will be approached for consent to undergo MITS (see [3.3 Inclusion Criteria](#_vy5r1r1cah0z) and [3.6 Representativeness of MITS Cases](#_7c3vdqt0d3d7)). CHAMPS Network sites will be expected to meet targets to identify a high proportion of all under 5 deaths in a timely manner such that MITS can be completed, ideally within 24-36 hours of the time of death. MITS has only been validated against CDA in the first 24 hours after death, but theoretical acceptability for conducting MITS within this short time frame was good in the CaDMIA study (>75%, Maixenchs et al, Submitted). As such, all efforts should be made to complete MITS within 24 hours of the time of death.

### Facility-based Surveillance

Each participating health facility at each site will be charged with obtaining a complete enumeration of deaths of children under 5 and stillbirths occurring within their facility. Healthcare-facility-based death notification should occur within a time frame that would allow completion of the MITS procedure within 24 hours. The timeframe may be extended if adequate refrigeration of the body is available and/or if subsequent evidence validates MITS after 24 hours, but all efforts should be made to complete the procedure within 24 hours.

The site PI and staff, in consultation with the CHAMPS PO, will pre-determine which health facilities will participate in CHAMPS mortality surveillance within each surveillance site. Within each facility, all wards or departments where child deaths or stillbirths may occur must be identified, and the entry points for labor and delivery, neonatal intensive care, pediatric admissions, morgue and any other areas of the facility which care for children must be mapped. The CHAMPS mortality surveillance team will establish daily presence at these points of entry and screen all potential cases for eligibility. The death-notification system must identify deaths that occur during shift changes or after normal working hours, in case the facility is not fully staffed 24 hours. Facility staff should be informed of the study and provided routine updates through staff meetings, sensitization sessions, and specific training sessions as appropriate.

CHAMPS surveillance staff will be responsible for daily enumeration of child deaths and stillbirths notified from the facility, and supervisors will monitor and troubleshoot as needed to ensure CHAMPS is notified of >80% of eligible child deaths and stillbirths in a time frame allowing completion of MITS procedures ideally in less than 24 hours. For quality improvement, these indicators will be jointly monitored by the CHAMPS site and the CHAMPS PO, and technical assistance will be offered as needed.

### Community Based Surveillance

Surveillance sites will be charged with obtaining a complete enumeration of deaths of children aged under 5 years and stillbirths within the catchment area (usually a DSS area), with a minimum of bias. For capture of under-5 deaths and stillbirths in the community, sites will use a combination of study staff and community sources (e.g., HDSS staff, pregnancy/neonatal surveillance staff, community/village recorders, community health workers, traditional birth attendants, obstetric care providers and if applicable, mortuary attendants, or other religious/community leaders) to enhance ongoing DSS systems to identify pregnancies, live births, stillbirths, neonatal, infant, and child deaths, ideally within 24 hours of death. A system of f these community reporters (e.g. 100-200, depending on the total population to follow) should be established to cover the surveillance population/catchment area. Each community reporter will be provided with a cell phone and consistent airtime to enable them to notify under-5 deaths and stillbirths in real time via SMS or phone calls to a central location that monitors these reports on a round-the-clock basis. The organization and logistics of the system to provide this coverage will be specified in the site mortality surveillance SOP and the site-specific protocol addendum. The community reporters will be trained on mortality surveillance eligibility criteria and will pre-screen for eligibility (child’s residence in surveillance area, age, time of death).

Importantly, before community deaths (as opposed to hospital deaths) may become eligible for MITS procedures, the study team will review the initial results of planned social and behavioral work (described in a separate protocol). The initial activities of the social and behavioral work will focus on any additional logistical and organizational arrangements to conduct the mortality surveillance, including investigation of methods and timing of approaching parents/guardians of recently deceased children for consent regarding MITS; optimal approaches for deaths detected in the afternoon or evening, and questions around transport of bodies from the household of the family to the location where the MITS procedures would occur.

## 3.3 Inclusion Criteria

#### 3.3.1 Mortality Surveillance

Overall mortality surveillance includes notification of death and collection of limited demographic and health data regarding the deceased child, clinical data on child and mother, and verbal autopsy. Inclusion criteria are as follows:

- Any stillbirth (meeting definition in this protocol) or child death under the age of 60 months
- Was the child (or in the case of stillbirth or neonatal death, the mother) considered a usual resident of the CHAMPS catchment area? (defined in site-specific protocol and SOP)
- Death occurred subsequent to initiation of CHAMPS mortality surveillance at the site

#### 3.3.2 MITS

A subset of deaths (ideally as many as possible, but initially a minimum of 50 per site) reported to the mortality surveillance system will be eligible for collection of MITS specimens, further clinical data and/or verbal autopsy as follows:

- Death meets inclusion criteria for overall mortality surveillance
- Consent and willingness to participate of parent, guardian or next of kin

## 3.4 Exclusion Criteria

#### 3.4.1 Mortality Surveillance

- Does not meet inclusion criteria for overall mortality surveillance

#### 3.4.2 MITS

- Death or stillbirth reported to the CHAMPS surveillance team >36 hours after death
- Unable to obtain consent for procedures
- Cases in which legal regulations (i.e. non-accidental or intentional death) preclude MITS procedures and CHAMPS data collection
- Body has been buried, cremated, or embalmed

## 3.5 Sample Size Considerations

### Mortality Surveillance

All notified under-5 death cases in the defined surveillance area will be enumerated and will be approached for consent for consent for verbal autopsy and abstraction of child and maternal clinical information. Given an under-5 mortality rate of approximately 50/1000 live births and a catchment area of approximately 3000 live births/year, we expect at least 150 notified under-5 deaths per year in addition to stillbirths.

### MITS

As an ongoing surveillance activity, sample size for CHAMPS MITS procedures will be determined by available resources and community acceptability, not by theoretical considerations or power calculations. Sites will begin with a target of 50 annual MITS cases and progressively increase, to a target of 200 or more per year, as resources allow, providing increasingly robust and precise estimates of cause of death in the study areas. We estimate that with a sample size of 200 MITS cases per year, a site would be able to estimate the proportion of deaths due to causes with true prevalences of 5%, 10%, 15%, and 20% with a level of precision quantifiable in terms of 95% confidence interval half-widths of approximately 3.1%, 4.2%, 5.0%, and 5.6%, respectively. Upon collecting 600 MITS cases (over approximately 3 years), the corresponding expected interval half-widths reduce to 1.8%, 2.4%, 2.9%, and 3.3%.

Each site will begin CHAMPS activities by performing MITS sequentially (so as to avoid any selection bias) on any eligible under-5 deaths and stillbirths for which they obtain consent for the procedure. Over time, sites may eventually detect many more under-5 deaths from their catchment areas in a year than it would be feasible to conduct MITS. Should that occur, sites will develop a defined sampling scheme (designed to avoid any selection bias) to determine which notified case should be approached for MITS procedures. (See section [3.6 Representativeness of MITS Cases](#_7c3vdqt0d3d7) below.)

The main endpoints of mortality surveillance include mortality rates, stillbirth rates, crude death rates, and cause-specific mortality rates, as described in [8.1 Data Analysis Plan](#_3n1e5xklj3g4).

## 3.6 Representativeness of MITS Cases

During the first year of surveillance at each CHAMPS study site, it is anticipated that primary attention will be given to obtaining as many MITS as possible within resource constraints while optimizing logistics (e.g., getting a sense of rates and reasons for acceptance and refusal, understanding challenges in obtaining access to facility-based vs. community-based deaths). As the number of MITS to be conducted will be opportunistic, no sample size adjustments will need to occur in relation to acceptance rates.

After the first year of data collection, the CHAMPS site study team (in collaboration with the CHAMPS PO) will analyze the initial data on deaths at each site in order to recommend measures aimed at making deaths selected for MITS as representative as possible within resource constraints, and/or to recommend differential sampling of certain age groups and/or types of deaths in order to optimize the resources allocated for MITS.

## 3.7 Recruitment

Once a death is notified to the CHAMPS site, a response team including trained health workers (according to predefined study SOPs) will be dispatched to the facility or community as soon as possible with a vehicle that can accommodate a recumbent child’s body if necessary (i.e., for non-facility deaths). One member of the team will approach the parents, guardian, and/or other family members to offer condolences and request consent for participation in mortality surveillance procedures: MITS (as soon as feasible within 24-36 hours of death), verbal autopsy (within 2-4 weeks of death), and clinical data collection (within 2-4 weeks of death). Trained study staff may also offer locally appropriate, supportive care and/or counselling to parents or family members as feasible during the initial contact during the period of grief. If parents provide consent for MITS, deceased children will be transported to a designated facility (if death occurred in the community) for collection of post-mortem specimens. A member of the family may accompany them to the facility if culturally appropriate. Formative research at each site (submitted in a separate protocol) will explore the need for a family or community representative to be present for the procedure itself. Immediately after performing the MITS specimen collection procedure (anticipated duration less than 90 minutes), the family will be offered transport of the child’s body back to the location requested by the family (within the CHAMPS catchment area), or preservation in the morgue refrigerators until the family is ready to accept the transport back to the household or location of the funeral. When appropriate, for cases approached to be part of the study and for whom parents or legal guardians choose not to give consent for the MITS procedure, transport may also be arranged by the study staff to a location requested by the family (within the CHAMPS catchment area).

Refusals and reasons for refusals will be documented and monitored at least weekly. For monitoring the quality of the team’s respectful interactions during this sensitive period, a different group of CHAMPS surveillance staff may subsequently revisit a sample of parents after the mourning period (those who consented as well as those who refused) for interviews regarding the team’s conduct. Ultimately families consenting to MITS will be compared with families which refused or were not notified in time, to ascertain systematic biases revealed by differences in geographic, demographic, clinical, or syndromic characteristics. Enumeration lists of eligible child deaths and MITS refusal reasons will be reviewed daily and weekly, with the aim of optimizing consent for MITS and achieving the study targets.

# 4. Study Procedures/Methodologies

## Mortality Surveillance

Initially, basic information will be collected about all notified under-five deaths and stillbirths, such as age, contact information (so the family may be contacted for further consent and data collection), and brief circumstances of the child’s death (2-3 sentence narrative). Of note, this is different from the verbal autopsy procedure to be conducted several weeks later and will serve only to orient the study team to the circumstances of death. Parents/guardians will also be approached for consent for other data collection activities, as described in sections [3.7 Recruitment](#_eqtkv5xbdiw8) and [11.1 Informed Consent](#_hf789nc6ydas)

## 4.1 Minimally Invasive Tissue Sampling Procedure

**Tissue Sample Collection**

After the parent or guardian has consented to the MITS procedure, teams will collect small tissue specimens ([Figure 1](#_cotnxq7l7u3d)) from the deceased child, using biopsy needles under sterile conditions. Teams will consist of 2-3 members, which may include pathologists, pathology technicians, physicians or clinical officers, nurses or specifically trained MITS technicians. Photographs and standard anthropometric measurements described below will be taken prior to specimen collection. Samples from the following organs will be collected: lung, heart, brain, liver, bone marrow, spleen or kidney from abdominal approach and, skin lesions if present. Details of this procedure are outlined in [Appendix 1 (Laboratory and Diagnostics Plan)](#_bh8fdwkyly4d) and may be modified slightly as needed based upon site requirements and experience. The specific and standardized set of samples collected and laboratory testing conducted with the MITS procedure will be re-evaluated periodically and may be modified based upon diagnostic utility, cost-effectiveness (which will include the consideration of time required to collect the specimens), and available resources.

For stillbirths and neonatal deaths, photos and specimens from the placenta, membranes, and umbilical cord will also be collected whenever feasible. Additionally, full-thickness 2 cm^2^ biopsies from the middle third of the placental disk will be obtained. Macroscopic evaluation of the placenta will also identify placental abnormalities (e.g., placental abruption, vasculitis, or infarcts). A 2 cm strip of the membrane and a 4 cm segment from the middle of the umbilical cord will also be collected.

**Non-tissue Samples**

In addition to the tissue samples, the following non-tissue samples will be collected during the MITS procedure (see specific and detailed methods in annex 1):

- Blood will be collected via subclavian or cardiac puncture
- Nasopharyngeal and oropharyngeal swab specimens
- Stools will be collected using a stool collection device (rectal forceps or similar) via the rectum. If whole stool cannot be collected, a rectal swab will be taken.
- Cerebrospinal fluid will be collected into a sterile tube.
- Urine may be collected by catheterization or suprapubic aspiration

**See Appendix B: Lab and Diagnostics Plan for details of specimen collection, processing, diagnostic assays, laboratory testing and quality management.**

### Safety

Standardized infection prevention and control precautions for the study personnel will be used for any contact with the body or body fluids. Elevated levels of personal protective equipment will be indicated for specific cases of increased disease transmission risk, such as suspected viral hemorrhagic fever. An SOP for infection prevention and control precautions will be developed by CHAMPS PO in collaboration with site investigators. HIV post-exposure prophylaxis will be available and provided to study staff by the study as needed in case of high-risk exposures (e.g., needlestick injuries).

### Figure 1. Illustrative examples of liver and brain tissue samples from a MITS procedure.


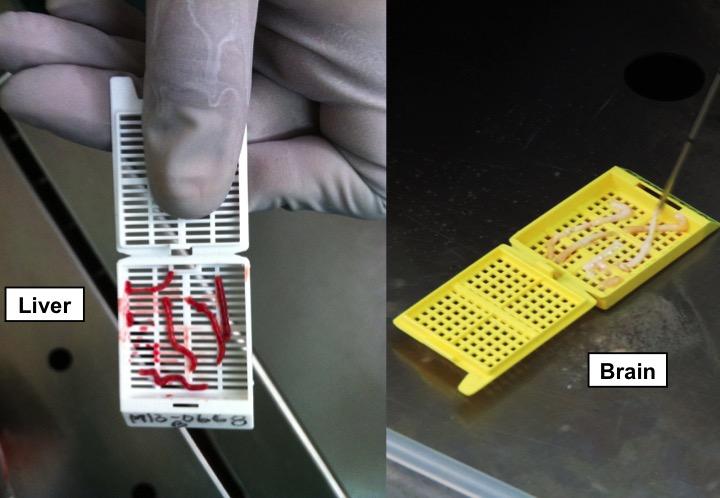


## 4.2 Clinical Data Collection

Consent will be sought to obtain all pertinent clinical records belonging to the deceased child as well as relevant maternal health information, whenever applicable. This will include abstracted medical information from health centers or hospitals consulted during the course of the terminal illness/event or prior contacts (including clinical and laboratory records); child health and vaccination cards, maternal health and pregnancy history, and other relevant data.

## 4.3 Photography and Measurements

As photographs and standard anthropometric measurements will be critical in detecting noninfectious causes of death, the response team will obtain consent from the parent or guardian to take photographs of the deceased child prior to the MITS procedures, and for conducting the aforementioned measurements (in the same location where the MITS will occur). Parents will be offered an opportunity to opt out of photographs as part of the general MITS consent process. All photographs will be taken following predefined SOPs (describing distance, lighting, angles and storage of files) and labeled using the barcoded card in the specimen collection kit that links the photos to the patient study identification number, and saved to a backup system at the site. The Birth Defects Surveillance Atlas of Selected Congenital Anomalies (<http://www.who.int/nutrition/publications/birthdefects_manual/en/>) will be used to guide diagnosis of birth defects. Quality control for these birth defect diagnoses will be conducted in a proportion of cases (~20%), using the support of a senior and experienced pediatrician. Length or height, weight and additional physical measurements will be recorded for complete data and to assess nutritional status. The physical measurements will include head circumference and middle upper arm circumference and will be defined in the MITS SOP.

## 4.4 Verbal Autopsy

With consent of the parents or guardians, all deaths in children <5 will be investigated with detailed verbal autopsies completed by caregivers of the deceased children, regardless of whether MITS was performed. We will aim to conduct verbal autopsies on deaths of all children aged less than 5 years within 2-4 weeks of the death. Trained interviewers will administer standardized verbal autopsy questionnaires, which are specifically designed to be delivered to the caregivers or family members of the deceased children. For children 28 days to <5 years, the standardized 2016 WHO Child verbal autopsy questionnaires will be used. For stillbirths and neonates (under 28 days), a CHAMPS enhanced perinatal verbal autopsy questionnaire (slightly modified from the WHO enhanced perinatal verbal autopsy questionnaire) will be used. The instrument has been pilot tested in perinatal mortality surveillance in Uganda (Saving Mothers Giving Life Initiative, Phase 2). Both verbal autopsy questionnaires will be adapted to the cultural context of the site, translated into local language and back-translated for accuracy. Both 2016 WHO Child VA and enhanced CHAMPS perinatal VA forms will include the optional narrative question to the informant early in the questionnaire. We will collect social and demographic information from the household in which the death occurs (e.g. DSS household demographic survey, social autopsy questionnaires), as feasible by the site.

## 4.5 Pregnancy and Neonatal Surveillance

Enrolling women in pregnancy surveillance early in pregnancy is needed to maximize timely identification of stillbirths and neonatal deaths and will favorably sensitize the community to CHAMPS activities given corollary benefits for pregnant women that would result from surveillance efforts (via pregnancy testing and enhanced promotion and access to augmented antenatal care). Social and behavioral work and community engagement activities (described in a separate protocol) will help guide design and implementation of pregnancy surveillance activities and community messaging. The CHAMPS Network will enroll women in pregnancy surveillance through a combination of identification via antenatal care (ANC) visits at health facilities and identification through community-based reporters. Pregnancy surveillance will be phased in at each site following formative research and piloting activities. A separate CHAMPS Network pregnancy surveillance protocol will be developed and submitted for all appropriate ethics approvals prior to implementation of this activity.

The NPHI, Ministries of Health, and other appropriate authorities will be actively engaged to promote provision of at least minimum standards of clinical care for pregnant, postpartum women and children under-5, following WHO and appropriate national and local guidelines.

**Key Pregnancy Surveillance Objectives**

1. Identify all pregnancies within the surveillance area.
2. Monitor all identified pregnancies in the surveillance area through birth. Whenever feasible, provide appropriate technical assistance and advocacy to help pregnant women receive minimum standard of care
3. Identify all deliveries and their outcome (stillbirth, live births)
4. Monitor status of babies through 2 months after live birth to identify neonatal deaths
5. Determine gestational age through ultrasound when feasible, as accurately as possible, to diagnose prematurity. Ultrasound estimation may be supplemented by clinical examination as appropriate or when ultrasound is unavailable.

**Secondary Objectives**

1. Establish baseline maternal clinical status (blood pressure, nutrition, anemia, HIV/syphilis/malaria status) and treat according to country guidelines
2. Establish maternal clinical status at time of facility delivery and store maternal samples per lab and diagnostics protocol.
3. Collect anthropometric data on all deliveries (including birth weight, length, head circumference)

## 4.6 Demographic Surveillance

Each CHAMPS Network site will ultimately run and maintain a demographic surveillance system (DSS), consisting of a minimum birth cohort of approximately 3000 live births (approximately 100,000 people). Having in place a complete, accurate, and well maintained DSS at each site will be essential for a thorough understanding of the populations in which CHAMPS operates and tracking changes at those sites. It will be necessary that all sites have current information on: the size of the population (overall, as well as numbers of women of reproductive age and children under 5), numbers of births annually, and numbers of deaths (by age) annually, and in- and out-migration rates. This information will allow up-to-date information on key population indicators and characteristics.

Data to be collected from households in the DSS area and standard procedures for data collection will be described in a separate protocol subject to appropriate local approvals. The CHAMPS team may provide technical and resource assistance as appropriate to sites in developing or modifying demographic surveillance procedures to meet CHAMPS requirements. Household and other epidemiologic data from the DSS system will be abstracted as part of the investigation of under-5 deaths among residents of the DSS area.

## 4.7 CHAMPS Determining Cause of Death (DeCoDe) Panel Process

For child deaths in which MITS was performed, a panel of 4 experts (epidemiologist, pathologist, clinician, microbiologist) will use all available information from linked maternal data; child clinical data; household and individual demographic and epidemiologic data; verbal autopsy; microbiology; molecular biology (TAC); clinical diagnostics (HIV, TB, malaria); site histopathology findings; and CHAMPS pathology laboratory (CPL) histopathology findings to assign each case a cause of death. Subject matter experts (e.g., genetics, toxicology) will be sought for consultation as needed. It is clear that not all death cases will have complete data from all CHAMPS program components, so the DeCoDe panel will base their cause of death determination on all available data. It is also possible that in some cases cause of death may not be possible to determine with the available information. Deaths will also be grouped into broad categories for comparison with previous studies (e.g., ICD-10 chapter codes and common causes of under-5 mortality). A standardized protocol for assigning cause of death considering all sources of evidence available will be developed with input from the SAC.

As depicted in Figure 2 below, individual panel members will review the case and assign immediate, associated and underlying causes of death using international cause of death coding standards, and will grade the causes from 1-5 with 5 representing the highest certainty and 1 representing the lowest certainty of death assignment, similarly to what has been done in the CaDMIA project (Castillo et al, submitted).All panel members will receive training on international cause of death standards. As a group, the panel will come to a consensus on the cause of death and note any disagreements on cause of death, with minority opinions recorded. The initial individual assignments of cause of death will be recorded, and the degree of agreement will function as another proxy for confidence, even after discussion and a consensus opinion.

The site-based panels will review all cases, and a central, Atlanta-based panel will review approximately the first 25-50 cases from each site and then a proportion (~10%) of randomly selected cases thereafter. The proportion of cases reviewed by the Atlanta panel may vary depending upon available resources and the observed degree of concordance with site panel cause of death determinations, as monitored over time. The central panel will include non-project staff members, configured with similar expertise as the local panels. Data will be collected for each case reviewed to identify ways that the process can be improved, as well as, potentially to help with training of members of future panels when new sites are activated.

Cases in which no MITS is performed will be subject to a simplified procedure, pending resource availability. Verbal autopsy data from neonates may be analyzed by physician coding, while VA data from deaths occurring after 28 days of life may be analyzed by an automated method (e.g., Tariff 2.0 or InterVA). For cases with available clinical data, one or more physicians may then assess the VA data and/or output from the automated coding software as well as available clinical data to assign a cause of death, similar to the DeCoDe process described above.

***Figure 2.*** ***CHAMPS Local Expert Panel to Determine Cause of Death (DeCoDe)***


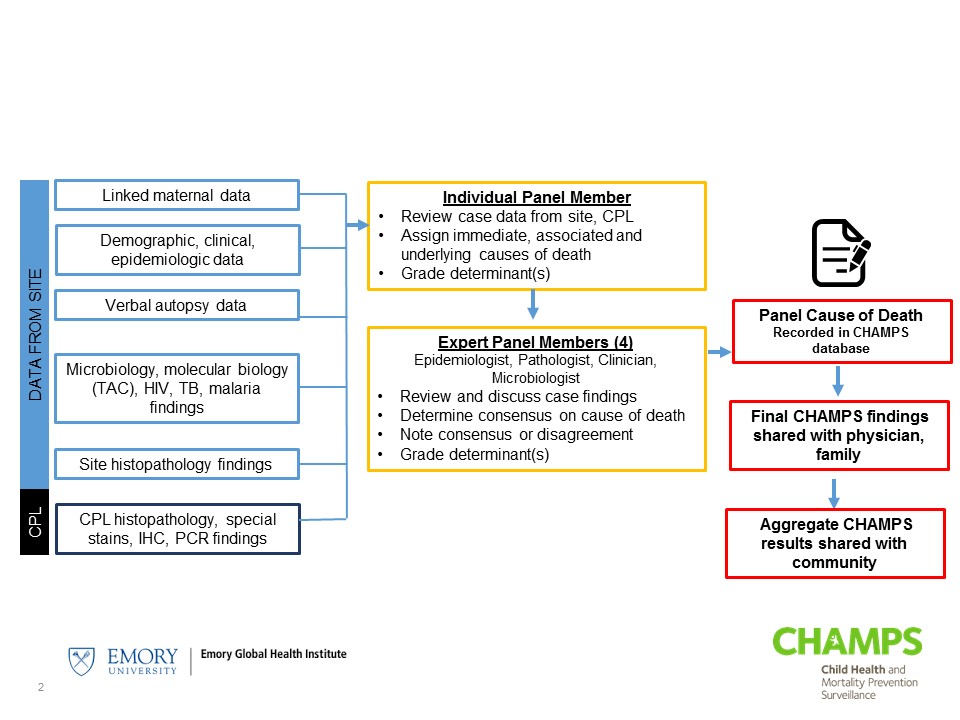


# 5. Training

## 5.1 Mortality Surveillance Procedures

A training workshop for surveillance workers will be conducted. This workshop will be coordinated by the site staff and the CHAMPS Program Office. Training modules will include: case selection procedures, assignment of CHAMPS ID, linkage and de-duplication of case data, enrollment and consent, confidentiality procedures, specimen collection, storage, transportation of specimens, universal precautions and proper use of personal protective equipment. Training will be monitored using competency testing for each component. All activities will be focused to allow qualified, fast and efficient implementation of the CHAMPS pathology protocol on site.

## 5.2 MITS and Diagnostic Procedure Training

MITS: A lead pathologist or clinician and technicians at each site will be trained on relevant facets of protocol implementation, and engaged in reading and interpreting specimens to enable local ownership and capacity development. All technicians and pathologists engaged in the MITS specimen collection, processing and evaluation will be trained, either locally or regionally. Training and monitoring for MITS and histopathology will be performed by ISGlobal through collaboration with the CHAMPS PO and the associated protocols.

TaqMan Array Card Assay: A five-day training workshop for laboratory technicians will be conducted at each site when all equipment is in place and functioning. This training will be conducted by technical experts from CDC and CHAMPS PO. Training modules will include: introduction to ViiA 7 or QS7 instrument and software, specimen preparation (pre-processing steps), nucleic acid extraction, PCR reactions and results analysis and interpretation. Additionally there will be training videos distributed that provide greater detail concerning the TAC assay software and result interpretations.

Reporting: Appropriate study staff will be trained on study-related and notifiable disease reporting requirements, as established by SOPs.

CHAMPS sites will implement a quality management system (QMS) to ensure consistent quality of protocols, tests, data, and results reported. Analysis and interpretation of MITS histopathology training will occur as part of procedural training and will also be continuous through the routine telepathology review of slides between sites and the CPL.

# 6. Storage and Archiving of Specimens

The vision for the CHAMPS Network specimen archive is to create a standardized, reliable, quality management approach to the collection, storage, access, and use of CHAMPS Network specimens with equitable governance by the global public health community. Supporting high-quality specimen collection, maintaining long-term specimen integrity, preserving patient confidentiality and privacy, and establishing robust governance procedures are core objectives.

Starting during the first 3-year phase of the project, maintaining the collection of archived specimens will allow further testing of specimens from cases in which an aetiology could not be established using study-defined diagnostics. In the longer term, the specimen archive will also serve as a resource to evaluate future technologies that will allow powerful diagnosis, pathogen discovery, assessment of host and/or pathogen genetic factors associated with virulence, epidemiologic and ecologic characterization, and a range of potential research applications. The informed consent procedure for MITS sampling will include information on the type of samples to be obtained, their planned uses and duration of storage.

Frozen tissues and non-tissue specimens would be stored at -80^o^C at surveillance sites and under liquid nitrogen (-140^o^C) at a central facility. All tissue specimens processed will be stored as formalin-fixed paraffin blocks (FFPE) and slides. The location and key findings will be recorded in the CHAMPS database.

## 6.1 Governance

The CHAMPS Network will create a robust governance system to facilitate the use of those specimens, stored for a long-term duration within the centralized repository, by the global public health community. Specific consent for long-term storage and future usage of these samples will be requested as part of the IC. Information regarding how to obtain specimens for research purposes will be publicly available on the CHAMPS Network website. The CHAMPS archive will be managed by a Specimen Archive Steering Group (SASG) comprised of NPHI or MOH representatives from countries with CHAMPS sites, representatives of study PIs, and other public health professionals. Materials transfer agreements will be put in place between the site and the program office (responsible for the centralized sample repository) so as to regulate the transfer and future utilization of such samples. Every country with a site that is part of the CHAMPS Network will have a representative on the specimen archiving committee, nominated according to SOPs to be developed by the CHAMPS PO in consultation with the site PIs. The Steering Group will be responsible for designing and approving specimen access and management policies, and delegating review of specific research proposals by a technical panel. This technical panel will evaluate the quality of proposals and prioritize with respect to specimen inventory.

## 6.2 Sample Access and Use

A formal process will be developed by the SASG for obtaining approval of further research beyond the initial determination of cause of death. A key aim will be to ensure that data be open to global health community and accessible for quality control checks, which will be part of the data sharing agreement contract. An SOP will be developed by the SASG describing these procedures in further detail. Site representatives will play a key role in these decision-making processes.

Proposals for consideration for use of the specimens must include information on all of the investigators, specific aims of the study, background and public health significance of the study, research design and methods, clinical significance of expected results, qualifications of the investigators, period of performance of the study and funding for the study. Additional requirements may be determined by the SASG. A specific written consent will be obtained for collection, multi-year storage and future testing of archived specimens. Review of proposals for analyzes not included in the original protocol will also include review by an Ethics Review Board if required according to the consent forms for cases to be included in the analysis. Once approved and specimens are released, periodic updates will be required for the study to continue.

Specimens will be retained until the end of the CHAMPS project with the possibility of extension if deemed appropriate. Specimen destruction plans, when requested by authorities from the country for each site (or requests to site-specific ethical review committees to preserve specimens in perpetuity) will be created within 5 years of the end of the project. If individual participants request to the surveillance sites that their specimens be destroyed, the CHAMPS Network will ensure these specimens are removed from the archive in all locations.

# 7 Data Management and Network Security

## 7.1 Data Governance

CHAMPS is a network of partner sites. Membership in the network requires adoption of the CHAMPS Network data and system standards. Sites will share data with the CHAMPS Network Program Office on a timely and regular basis, as defined in the data sharing agreement and will allow data to be released to authorized users of the CHAMPS Network global data repository. The CHAMPS Network global data repository will be a centralized, web-accessible, composite database of CHAMPS Network data from all participating sites that will provide data visualizations and analyzes.

The CHAMPS Program Office will facilitate the creation of a Data Governance Committee (DGC) to determine overall data stewardship policies, including data quality issues and policies to foster responsible data sharing. The DGC will be primarily composed of members that are external to the CHAMPS PO that have strong expertise in epidemiology, pathology, informatics, statistics, surveillance research policies, as well as those who hold a key interest in CHAMPS data such as Ministries of Health and NPHIs. Site representatives (e.g., site Principal Investigators) will play a key role in the formation and policies of the DGC. In addition, the DGC will engage global health partners that can most effectively use CHAMPS Network data to reduce childhood mortality.

Approaches and guidelines for data clearance, access, and use will be developed by the DGC in collaboration with the CHAMPS PO, the SACs, site directors, MOH leaders and program leadership at the BMGF. Access to the data will be role-based and subject to approval via a process defined by the DGC. Users will be granted differing levels of access and manipulation depending on role and data grouping. The emphasis will be on making high-quality data available as soon as possible after initial collection, and it is anticipated that de-identified data sets may be made available for download following a simple registration process and approval. Data sets that contain fields that may identify a specific patient (e.g. photographs, date of death, service dates, date of birth) would be subject to additional protections and would be released only to investigators with a demonstrated research need after a formal approval process specified by the data governance committee. Any data access would require a user to complete a registration process. The registration process would allow the CHAMPS PO to monitor how data are being used and how many users have downloaded each data type. The CHAMPS data governance structure will define the rules of engagement governing timing and access to data.

## 7.3 Data Entry, Transmission, Security and Backup

CHAMPS datasets captured at each CHAMPS Network site will be maintained at each site in a local secure data repository as well as mirrored in a secure global data repository maintained by the CHAMPS PO. Any physical data collection instruments containing unique identifiers will be stored in closed, locked filing systems with restricted access at the CHAMPS study office. Sites will transmit data to the secure global data repository on a routine basis. For most types of data, ideally, we expect data will be gathered locally via paper or electronic capture and communicated to a site’s headquarters office within 7 days of data capture/recording. Once data have passed local quality assurance (QA) procedures and are validated in the site’s data repository/database, they will be communicated to the CHAMPS Network PO following appropriate QA and standard data transmission protocols. The projected time from electronic data capture to data transmission to the CHAMPS Network global data repository would range from 14 to 30 days. There will be additional QA cycles performed at the PO prior to making curated data available for analysis. This timeframe is a projection based and may change with actual experience based on variance in time to complete appropriate level of quality control on data prior to submission to the PO.

All CHAMPS sites and the PO will have reliable data management and storage infrastructure that follows relevant guidelines contained in the ISO 27001 specification and/or any required in country standards with appropriate levels of security, redundancy and backup.

The CHAMPS global data repository managed by the PO will comply with Emory University information security standards. Local site repositories will be asked to comply with PO information security standards, though local regulations and standards may take precedence if applicable.

## 7.4 Enrollment and Unique Identifier

A unique CHAMPS identifier will be prospectively assigned to each subject/person linking all surveillance records as well as all specimen collection data to an individual subject/person. The CHAMPS identifier will be unique across all subjects and all sites. The CHAMPS identifier will be used on all forms and samples to enable linking data for a specific subject across data sets in the absence of personally identifying information. Only authorized study staff will have access to keys linking CHAMPS identifiers to personally identifying information.

## 7.5 Data Collection Instruments

CHAMPS sites will collect data as described in section [4. Study Procedures/Methodologies](https://docs.google.com/document/d/1JoByfRSQGTKgPTa87W2mLpFcN05RV58qIVSbasAnU98/edit#heading=h.se23pz2qcepw). Standardized forms will be developed as described below and adapted to local language and context, in a manner allowing aggregation and and analysis across sites and years. For example data elements, see [Appendix 3](https://docs.google.com/document/d/1JoByfRSQGTKgPTa87W2mLpFcN05RV58qIVSbasAnU98/edit#heading=h.9kal5pc9wx8j).

**Death Reporting**

- Initial death notification form (death reports may be completed by a variety of CHAMPS designated staff such as community members conducting mortality surveillance, pregnancy surveillance, or DSS in the community, or clinical staff conducting mortality surveillance in the health facility) including brief circumstances of death (e.g., accidental vs. natural).
- Eligibility screening form (for both health facility, community-based efforts)
- Consent withdrawal and reasons for consent refusal

**Clinical and demographic abstraction forms**

- Clinical abstraction form for deceased child’s terminal illness (from clinic, hospital or other health care setting)
- Photographs of child health and vaccination cards
- Demographic surveillance data abstraction
- Household health utilization survey abstraction
- Maternal linked clinical data abstraction (e.g. obstetric history)

**MITS**

- MITS case report and specimen collection and results forms
- Digital photography form
- Disposition of body form

**Verbal autopsy**

- 2016 WHO verbal autopsy questionnaires for deaths of children aged four weeks to 11 years, and for deaths of children aged under 4 weeks.
- CHAMPS Enhanced Perinatal Verbal Autopsy (used in cases of stillbirth as well) (see section [4.4 Verbal Autopsy](https://docs.google.com/document/d/1JoByfRSQGTKgPTa87W2mLpFcN05RV58qIVSbasAnU98/edit#heading=h.u0vbqaj2s1pn))
- Verbal Autopsy Cause of Death Determination Form

**Panel Determination Forms**

- DeCoDe Panel Member Individual Cause of Death Determination Form (and additional related ICD-10 translation form)
- DeCoDe Panel Consensus Cause of Death Determination Form (and additional related ICD-10 translation form)
- Preliminary information and final results to be communicated to family forms

**Data reporting**

- Aggregate site and global summary data reporting forms (may be electronic format)

## 7.6 Data Collection, Editing and Management

The CHAMPS PO will provide the sites with the standard CHAMPS specifications for data collection forms, specimen data management, specimen shipment, laboratory results, and other data needs as required. The CHAMPS PO will also provide specifications for data formats and models for submission of CHAMPS data to the PO. Sites that have existing systems to support data collection that conforms to CHAMPS specifications may choose to utilize their existing systems. Additionally, data collection implementations may vary across sites for specific types of forms or data with some sites choosing to do all field data collection with paper followed by entry into an electronic system at a central location while others may utilize electronic field data collection on mobile devices. SOPs will be developed and adapted to site contexts and a CHAMPS PO technical assistance team will work with the sites prior to activation of data collection activities to ensure any adaptations are consistent with CHAMPS specifications.

General procedures/expectations:

- Any form-based data that is captured via paper will be transcribed into an electronic data capture system and should include double data entry to ensure accuracy and completeness. Double data entry may not be possible for some existing laboratory information systems, but other means of ensuring accuracy for entry of paper based data may be utilized, such as barcoded labels on specimen manifests, configured workflow with data templates and business rules in the laboratory system that minimize assignment of erroneous specimen metadata for specific specimen types.
- Data validations will be incorporated into any electronic data capture to enable validation at time of entry.
- All data capture systems should support audit trails that provide audit entries for record creation, update, delete and views/read.
- Data submitted to the PO will conform to CHAMPS network data submission specifications including use of standard vocabulary/lookup values, variable names/sequence, data set structure and file types.

## 7.7 Quality Assurance and Quality Control Measures

Data will be collected at each site and aggregated locally, including prior to submission to the CHAMPS PO. A continuous data quality process will be followed at the local site and then again at the PO when data are received. The process will include the execution of data quality scripts and generation of quality reports at the local site and at the PO. All issues identified by the site will be corrected before transmission of data to PO. If issues are found in PO QC, reports with all issues will be sent to the site for correction QC and resubmission of data. Sites may manage local dissemination to local stakeholders and partners. The CHAMPS PO will manage data across CHAMPS sites and provide managed access to that data as well as cross site analysis of data. The projected timeframe from initial capture of data to loading to the CHAMPS PO repository for further cleaning is project to be between 15 and 30 days.

General principles related to QA/QC (site specific procedures will be specified in SOPs) for CHAMPS data will include:

- 100% QC on all forms by supervisor prior to data aggregation and automated QC
- Sites will perform QA/QC on data via manual and automated processes. Rates/statistics on any errors detected and corrected during site QA/QC will be recorded and reported to the PO along with resolution times for errors. Resolution time will typically be measured in the number of QA/QC cycles required to correct a given error.
- The PO will perform QA/QC on data received from sites via automated processes. Error reports will be provided to the sites for required remediation and resubmission of data. Rates/statistics on any errors detected and corrected during site QA/QC will be recorded and aggregated along with resolution times for errors. Resolution time will typically be measured in the number of QA/QC cycles required to correct a given error.
- Procedure for verifying that cases meet case definitions may be performed via manual or automated processes using scripts or data analysis techniques.
- Deduplication of persons/subjects will be performed via a combination of automated and manual processes. Automated processes will detect duplicates based algorithms flag any records with less than a 100% confidence level for uniqueness for any given subject. Records that fall below 100% confidence will be manually analyzed and corrected.
- Re-abstraction for QC of a small percentage of clinical records
- Re-classification of cause of death by separate panel for some percentage of deaths

# 8. Data analysis

## 8.1 Data Analysis Plan

Analyses of CHAMPS data will be conducted to comprehensively describe overall and cause-specific under-5 mortality at each site and across sites. The data elements collected will allow for calculation of overall and cause-specific mortality for various conditions and age groups (e.g. diarrheal disease, respiratory illness, febrile illness, stillbirth and neonatal mortality) in the study population and will allow for the consideration of a variety of contributors to death, whenever available, including levels of illness severity, delays in accessing care, and healthcare access, host factors (including malnutrition and inheritable diseases, like hemoglobinopathies), co-infection contributors to death, and environmental exposures. These analyses will be conducted at sites and at the CHAMPS PO using commonly available software packages, such as SAS, STATA and Microsoft Excel. Specific measures will include but not be limited to the following and may be stratified by significant comorbidities (e.g. HIV status). Site-specific analysis plans will be described in the site-specific addenda to CHAMPS protocols, and the PO analysis plan will be described in a separate protocol describing CHAMPS activities conducted by the PO.

**Key analyses**

Specific methodologies used for analyses will be developed in consultation with a variety of experts and will depend upon population data available for each site. Examples of key analyses to be performed include calculation of stillbirth and under-5 mortality rates.

Life-table methods will be used to calculate the following:

- **Mortality rates (per 1000 live births per year, calculated using life-table methods):**
  - Under-5 mortality: number of children who die by the age of five years, per 1000 live births
  - Infant mortality: number of children who die by age of 1 year, per 1000 live births
  - Neonatal mortality: number of children who die by age of 28 days, per 1000 live births
- **Stillbirth rates**
  - Number of stillbirths (>1000 g birth weight or 28 wk EGA) per 1000 total births.
- **Crude death rates per 100,000 person-years (using life-table methods)**
  - Number of children who die by the age of five years, per 100,000 person-years of observation
  - Infant mortality: number of children who die by age of 1 year, per 100,000 person-years of observation
  - Neonatal mortality: number of children who die by age of 28 days, per 100,000 person-years of observation
  - Stillbirth rate: number of stillbirths >1000 g birth weight per 100,000 person-years of observation
- **Estimated cause-specific mortality rates** for each site (per 100,000 person-years). The primary CHAMPS target is to complete all of the procedures to arrive at a cause of death determination on >80% of MITS cases within 4 months of the child’s death.
  - Analysis of distribution of causes of death for site and across all sites, showing proportion of all deaths in DSS area represented by each cause (ICD-10 chapter headings and specific diagnoses or other common categories, such as those used in the Global Burden of Disease publications (IHME 2013; Wang et al 2014a; Wang et al 2014b)
- Cause- and pathogen-specific incidence rates: modeled by extrapolating syndromic cause of death fractions from available data (e.g., causes of death determined by verbal autopsy, clinical data abstraction and specific pathogens identified by MITS) to the total population under surveillance.

Other analyses may include:

- Multivariable analyses may be conducted using demographic, maternal and laboratory/histopathology data to explore risk factors for death or specific conditions among children and their families residing within the CHAMPS catchment areas.
- Analyses of key infectious cases of death for the purpose of detecting and responding to outbreaks of known or novel pathogens (see annex 1 for specific methods for pathogen screening and detection)
- Comparisons of cause-specific mortality fractions as determined by verbal autopsy, clinical data, MITS, and combinations of these data sources.

***Analysis for Meeting Reportable Disease Standards***

Laboratory diagnostic data collected by CHAMPS may provide confirmation of infectious disease pathogens important for timely public health action. Site investigators will identify pathogens in the CHAMPS laboratory and diagnostic plan that are also notifiable diseases by the public health authority. Laboratory-based surveillance alerts and reporting will be implemented to meet notifiable disease reporting timelines using SOPs. Since CHAMPS has the potential to augment laboratory diagnostics beyond a currently available standard, SOPs will also ensure prompt reporting to the public health authority of diseases identified by international disease surveillance normative guidance, such as the [*Technical Guidance for Integrated Disease Surveillance and Response in the African Region, 2nd edition*](http://www.cdc.gov/globalhealth/healthprotection/idsr/pdf/technicalguidelines/idsr-technical-guidelines-2nd-edition_2010_english.pdf)*.*

Prospective mortality surveillance may identify clusters of under-five deaths or likely reportable disease syndromes. Site investigators will ensure SOPs allow for regular reporting of death clusters and suspected notifiable disease syndromes to the public health authority. This may be achieved by integration of CHAMPS study staff in recurring health authority surveillance data reviews.

***DeCoDe Panel Data Analysis***

The CHAMPS PO, working with modeling experts will develop models to attribute cause of death, and through consultation with a group of technical experts, a hierarchy will be developed to determine which data are most reliable and relevant and should be given the most weight for cause of death rate calculations. In the case of immediate deaths from traumatic causes such as drowning or motor vehicle accidents, enhanced verbal autopsy may be sufficient to accurately attribute cause of death; evaluation of specimens from these deaths would provide immensely valuable comparison (control) material which will aid in interpreting the data from all other MITS, particularly in relation to association of causality to pathogens detected by the microbiological procedures. The models will use pre- and post-mortem data, including clinical and basic laboratory data and findings from microbiologic and molecular investigations from severely ill children (where available) and children who died (including histologic findings from the subset of deaths for which post-mortem specimens are collected), covariates that have been shown to influence child survival (e.g., skilled birth attendance, maternal literacy, immunization), as well as other epidemiologic and demographic information. As datasets are built from CHAMPS Network sites, models will be continually refined and applied to cause of death estimates.

## 8.2 Limitations

Anticipated limitations of CHAMPS data include lack of generalizability of facility-based MITS data prior to establishment of the site DSS area, as such cases will not necessarily constitute a representative sample of deaths in the catchment area; limited generalizability to areas outside the DSS area, as the DSS area may not be representative of the rest of the host country or other similar DSS areas; limited direct utility of MITS for determining certain causes of death (e.g. trauma and other non-infectious causes of death, and the potential of the MITS collection process of missing the location (i.e. the needle did not enter the area of the infection) of the mortality-associated pathogens). Formative research conducted prior to the initiation of and during CHAMPS activities (to be submitted under a separate protocol) will explore the existence of important cultural limitations on the procedure’s application in the community and suggest appropriate mitigation strategies. An important *a priori* limitation of the MITS procedure is related to the fact that localized organ lesions may not be appropriately sampled with fine needle “blind” biopsies. This is important as, at this time, no imaging techniques will be used to guide the MITS procedure or to localize lesions. Data indicate approximately 80% concordance between the MITS method and the CDA in the CaDMIA study (Castillo et al, Submitted).

# 9. Managing Unexpected or Adverse Events

## 9.1 Response to new or Unexpected Findings and to Changes in the Study Environment

An extensive socio-behavioral science and community engagement plan has been developed as a separate protocol, which will facilitate multifaceted bidirectional communication and exchanges with study communities, including monitoring of community and family concerns and perceptions of the program; addressing concerns, needs, desires, misperceptions and rumors proactively of various members of the community, as well as in-country stakeholders, outside the community, who may have interests in or concerns about CHAMPS, like political, health, religious, development, and other thought leaders and the media. Aggregate, de-identified summary data from CHAMPS will be reported back to the community and to identified in-country stakeholders on a regular basis, along with changes to the study methodology resulting from new or unexpected findings or health-related changes.

## 9.2 Identifying, Managing and Reporting Adverse Events

The CHAMPS Network is an observational study, not an intervention study. Specimens will be collected only from deceased children. Nevertheless, study-related adverse events involving surviving family members could be possible (e.g. inadvertent disclosure of PII, adverse psychological events related to study participation). Any unforeseen or unexpected adverse event (AE) that occurs in association with study procedures will be recorded. Safety events that the site PI deems to be unexpected, serious, and at least possibly related to study procedures will be reported to the site investigators, local IRB and CHAMPS PO. As this is a non-interventional surveillance study, sites will not report deaths of children or their family members to the site IRB, unless the death is considered to be unexpected, serious and at least possibly related to study participation or procedures.

In general, if an AE occurs, all attempts will be made to provide stabilization and clinical management to protect the safety and well-being of the individual. A Local Safety Monitor (LSM) will be identified prior to study initiation. Study staff will document the AE using a standardized reporting form, which will be sent to the LSM within 24 hours of site staff becoming aware of the event. The LSM will verify the occurrence and investigate circumstances of the AE and report it to the site PI or designee within 24 hours of verification. The PI or designee will determine seriousness, expectedness and relatedness of the event.

Events that the site PI considers to be serious, unexpected, and at least possibly related to study procedures will be reported to the site investigators, local IRB and CHAMPS PO within 48 hours of notification of the site PI of the event. The site will transmit the completed SAE form to the designated CHAMPS Network Safety Monitor located in the CHAMPS PO. The Safety Monitor will receive regular updates on each SAE event until resolution of the event.

The PO will prepare reports of all SAEs (across sites) by category, outcome, severity, relatedness to the procedure, and site on a monthly basis. The CHAMPS Network Safety Monitor summarize and assess SAEs and evaluate the need for protocol or procedural changes indicated by these data. The CHAMPS Network PI or designee will then make a final decision about whether and how to implement such changes.

# 10. Dissemination, Notification, and Reporting of Results

## 10.1 Notification of Participants of their Individual Results

One or more contacts with families will be made after the MITS procedure (e.g. to conduct VA and to update contact information as needed and maintain rapport with family while laboratory and pathology work is underway). Final cause of death will be communicated to family within approximately 4 months. Results will be communicated to the parent/guardian even in cases where the cause of death is unclear and may include negative tests as appropriate. Staff will attempt to address any questions that may arise.

Diagnoses (such as TB or HIV) with clear public health or family health implications should trigger appropriate local public health action (e.g. referral of family members as appropriate to the local health facilities). SOPs will be developed with each site to delineate these actions in collaboration with local and national public health authorities.

In addition, each site will assign one or more clinical monitors, whose function will be to assure that any significant clinical findings resulting from CHAMPS procedures are appropriately managed or referred with documented follow-up (e.g., previously unknown HIV infection of mother; active TB case in a household; or serious condition discovered on ultrasound during pregnancy surveillance procedures).

## 10.2 Notification of Communities of Study Findings

Aggregate and de-identified CHAMPS data will be shared with the community on a regular schedule in order to promote community awareness and understanding of CHAMPS efforts and findings and to facilitate community action, when appropriate, to address preventable causes of under-5 mortality.

If resources permit, sites may facilitate formation and activities of a Community Action Team (CAT), composed of community leaders, health systems representatives, and others to effect change in the community and health systems based upon findings and recommendations from case reviews. Established programs exist in the US and elsewhere successfully implementing this model (e.g., [NFIMR](http://www.nfimr.org/site/assets/docs/FIMR%20Manual.pdf), [FIMR-HIV](http://www.fimrhiv.org/), and others). The CHAMPS PO will facilitate provision of technical assistance to sites wishing to implement such a Community Action Team, and a small amount of seed funding may be available for CAT activities. Such activities will be defined in a site’s site-specific protocol and SOPs.

## 10.3 Anticipated Products Resulting from the Project and Their Use

Users will be able to review the epidemiologic trends to identify disease intervention opportunities and challenges, as well as differentiate disease burden within a particular site and demographic population. Finally, as the CHAMPS Network matures, it will produce a reservoir of pathogen information tied to geographies, ecologic considerations, and populations. CHAMPS Network data are anticipated to serve as a foundation for informing vaccine development strategies and policies for optimal use that will maximize their impact for prevention of childhood illness and death. CHAMPS Network provides unique opportunities to investigate populations and pathogens over time and locations. Proper organization and data collection will allow trends to be observed and predictors of childhood mortality to be tracked over decades. CHAMPS will also provide extensive technical assistance to the local NPHI so they are positioned to make optimal use of available data (from CHAMPS and elsewhere) to drive policy to prevent childhood morbidity and mortality.

## 10.4 Dissemination of Results

Publicly available data will be reported to relevant stakeholders regularly and may include aggregate data and analyses regarding causes of death in the community and de-identified case data for training and secondary analyses**.**

Results from the study may additionally be presented at scientific conferences and/or in peer-reviewed manuscripts to contribute to the body of knowledge of childhood morbidity and mortality. A publications committee will be constituted by the PO and sites to review proposed publications involving more than one site’s data. Sites will have similar mechanisms for reviewing proposed uses of site-specific data.

# 11. Ethical and Human Subjects Considerations

## 11.1 Community Entry and Engagement Strategy

Community leaders and community advisory boards will be identified, approached and informed about the study. Thereafter community meetings will be organized within the study areas to give community members the opportunity to ask questions and to raise any concerns about the study. Extensive ongoing community engagement activities will be conducted and will be informed by a detailed formative research and community engagement protocol, submitted separately.

## 11.2 Informed Consent

Parents or guardians of deceased children (at the hospital/health facility, or in the community) will be approached for written informed consent for participation of their deceased child in CHAMPS mortality surveillance procedures, following clear explanation of the study and opportunity to ask questions. Model informed consent forms in English for adaptation by sites may be found in [Appendix 2](#_aft65mjvyehk).

Formative research will identify whether there are other appropriate family or community members to be consulted about decisions when a child dies, as well as the form of any appropriate contributions to the family or community. All response team staff will be trained to interact respectfully with the family. For deaths in the community, the consent procedure will be undertaken by a designated and specially trained member of the response team. If the body must be transported to a health facility for the MITS procedure, a family member or trusted confidant may be requested to accompany the response team to the location. In some settings, depending on the formative research findings, the site’s ability to accommodate an observer, and the family’s wishes, this person may be allowed to be present during the MITS procedure.

Families or guardians who do not consent to MITS may be asked for consent for medical record abstraction and to be re-contacted within 2-4 weeks, at which point they will be asked for formally asked for consent for verbal autopsy interview.

The initial consent will cover the following procedures: transport of the body (if applicable for community deaths); photography, measurements, MITS specimen collection and storage (if eligible and parents agree); abstraction of child’s clinical records relating to the child’s illness; linkage of maternal data (if applicable for women previously enrolled in pregnancy surveillance); return visits at 1 month and 3-4 months to provide preliminary and final cause of death and re-contacting for future research. As required by ethics review boards at each site, consent may be separately requested for participation in the study; international shipping and laboratory testing of de-identified patient specimens (if MITS is performed); storage of de-identified patient specimens; and future genetic testing on specimens (both host and pathogen genes). Model informed consent forms in English for adaptation by sites may be found in Appendix 2. The site-specific forms must be translated into the applicable local language and back-translated into English and will be provided to the CHAMPS Program Office.

The protocol and informed consent forms will be reviewed and approved by site IRBs prior to any protocol-specified procedures being conducted. The investigator will regularly inform the site IRB as required about the progress of the study.

## 11.3 Risks and Benefits

### 11.3.1 Risks

Relatives will be grieving for the deceased child and may consider CHAMPS procedures to be intrusive on the grieving process, on the traditional burial procedures, or to have ramifications for cultural norms. Ongoing formative research in each CHAMPS site is currently exploring these issues with communities, bereaved relatives, and key informants. Findings from the formative research will inform this study to ensure the highest level of ethical responsibility and compassionate interactions are maintained. Other possible risks related to new diagnoses (post-mortem) of tuberculosis, HIV and/or other potentially sensitive contagious infections will be communicated and dealt with according to national standards, both in terms of management and preventive strategies for contacts of the deceased.

### 11.3.2 Benefits

Relatives will be informed that the procedures may provide information to them on the actual cause of death of the child. Not understanding why or how the deceased individual died has been identified through formative research to be a very important concern among relatives, the community, and health service providers. Formative research has also identified that, in the case of a child’s death, the mother may be blamed for having caused the death, and therefore information on a biological cause of death may be of interest. Study staff will personally inform relatives of the findings from study procedures, including the possibility that the cause may still be unknown. The precise nature of the feedback information to be provided (either verbal or written) and the specific information to be given (for example the “syndromic” nature of the death, or the specific pathogens detected, if they are found to have public health implications or lead to specific preventive actions at the household level) will be informed by the social and behavioral work, and agreed upon and specified in an SOP. There may be potential health benefits to family members in specific cases where HIV and/or TB are newly diagnosed or for instance, in the case of other infectious diseases (i.e. meningitis) that may require the administration of antibiotic prophylaxis to contacts. Communities through key opinion leaders (e.g. chiefs, elders, religious leaders) will receive anonymized, aggregate information on causes of death of all deaths undergoing MITS within the surveillance area in that country. Results of the study will assist health services to better understand the causes of death by location.

Depending upon findings of the formative research and upon practices deemed acceptable and helpful by the study team, each family may receive a contribution towards burial expenses paid directly to the undertaker. Formative research will be performed under a separate protocol to define adequate compensation that is not an undue influence on the decision-making. A study vehicle may be used to transport the body back to the home of the deceased after procedures are completed. All parents/legal guardians approached for consent for the MITS procedures will be offered (irrespective of their acceptance or refusal) the same compensation, which may include (again depending upon perceived optimal strategies at the site) free transportation of the body of their deceased child to their household (within the study area). Additional support for the burial will be defined locally according to acceptable ethical standards.

### 11.3.3 Voluntariness, Privacy and Confidentiality

Participation in this study is completely voluntary. Families of the deceased can deny permission to conduct the MITS or any other study-related procedures and can as well choose not to even speak with the study staff without being subjected to duress and without subsequent consequences.

All data will be stored securely, regarding both the names of those who have participated in the study and results of the MITS procedure. As noted, results will be translated so that they are interpretable by the family and will be given directly to next of kin; any wider dissemination of data will be anonymized and aggregated to ensure that results are not identifiable at the individual level.

All study staff and associated personnel (e.g. mortuary and autopsy assistants) will be oriented to confidentiality procedures, and identity of the individuals will be limited to the extent possible (e.g. limiting use of full name of the case during the procedure).

### 11.3.4 Public Safety Considerations

Any mortality reporting system may identify potential threats to public safety, whether manmade or environmental. CHAMPS sites will engage the community and local authorities on appropriate procedures for promptly reporting public safety concerns to the relevant authority. Initial death reports or the cause of death determination process may identify suspected non-accidental trauma with the potential for ongoing harm to others in the household. Site investigators will ensure that legal and health reporting requirements, as established by local authorities, are incorporated into study procedures.

# 12. References

Avrahami R, Watemberg S, Hiss Y, Deutsch AA. Laparoscopic vs conventional autopsy. A promising perspective. *Arch. Surg.* 1995;130:407-409. doi:10.1001/archsurg.1995.01430040069014.

Avrahami R, Watemberg S, Hiss Y. Thoracoscopy vs conventional autopsy of the thorax. A promising perspective. *Arch. Surg.* 1995;130:956-958. doi:10.1001/archsurg.1995.01430090042016.

Barcelona Centre for International Health Research (CRESIB). *Proposal: Validation of the Minimally Invasive Autopsy Tool for Cause of Death Investigation in Developing Countries*. 2012.

Bassat Q, Ordi J, Vila J, et al. Development of a post-mortem procedure to reduce the uncertainty regarding causes of death in developing countries. *Lancet. Global Health* 2013;1(3):e125-6. doi:10.1016/S2214-109X(13)70037-8.

Ben-Sasi K, Chitty LS, Franck LS, et al. Acceptability of a minimally invasive perinatal/paediatric autopsy: healthcare professionals’ views and implications for practice. *Prenatal Diagnosis* 2013;33(4):307-12. doi:10.1002/pd.4077.

Breeze ACG, Jessop F a, Set P a K, et al. Minimally-invasive fetal autopsy using magnetic resonance imaging and percutaneous organ biopsies: clinical value and comparison to conventional autopsy. *Ultrasound in Obstetrics & Gynecology.* 2011;37(3):317-23. doi:10.1002/uog.8844.

Boerma T. & Mathers CD. The World Health Organization and global health estimates: improving collaboration and capacity. *BMD Medicine*. 2015; DOI 10.1186/s12916-015-0286-7

Butler D. Verbal autopsy methods questioned. *Nature*. 2010; 467:1015.

Byass P. Usefulness of the Population Health Metrics Research Consortium gold standard verbal autopsy data for general verbal autopsy methods. *BMC Med*. 2014; 12:23.

Castillo P, Ussene E, Ismail M, et al. Pathological methods applied to the investigation of cause of death in developing countries: minimally invasive autopsy approach. *Plos One*. 2015; 10(6): e0132057.

doi:10.1371/journal.pone.0132057

Cox J a, Lukande RL, Kateregga A, Mayanja-Kizza H, Manabe YC, Colebunders R. Autopsy acceptance rate and reasons for decline in Mulago Hospital, Kampala, Uganda. *Tropical Medicine & International Health.* 2011;16(8):1015-8. doi:10.1111/j.1365-3156.2011.02798.x.

Fan JKM, Tong DKH, Poon JTC, et al. Multimodality minimally invasive autopsy--a feasible and accurate approach to post-mortem examination. *Forensic Science International* 2010;195(1-3):93-8. doi:10.1016/j.forsciint.2009.11.019.

Fligner CL, Murray J, Roberts DJ. Synergism of verbal autopsy and diagnostic pathology autopsy for improved accuracy of mortality data. *Population Health Metrics*. 2011; 9:25. doi: 10.1186/1478-7954-9-25: 25–29.

Lishimpi K, Chintu C, Lucas S, Mudenda V, Kaluwaji J, Story A, et al. Necropsies in African children: consent dilemmas for parents and guardians. *Arch Dis Child*. 2001; 84:463–467. 4.

Garenne M. Prospects for automated diagnosis of verbal autopsies. *BMC Med.* 2014; 12:18.

Gupta N, Bharti B, Singhi S, Kumar P, & Thakur JS. Errors in filling WHO death certificate in children: lessons from 1251 death certificates. *Journal of Tropical Pediatrics*. 2014;60(1): 74-8. doi: 10.1093/tropej/fmt059. Epub 2013 Jul 31.

Jha P. Reliable direct measurement of causes of death in low- and middle-income countries. *BMC Med.* 2014; 12:19.

Lishimpi K, Chintu C, Lucas S, et al. Necropsies in African children: consent dilemmas for parents and guardians. *Arch. Dis. Child.* 2001;84(6):463-7. Available at: <http://www.pubmedcentral.nih.gov/articlerender.fcgi?artid=1718810&tool=pmcentrez&rendertype=abstract>

Martinez MJ et al. Infectious cause of death determination using minimally invasive autopsies in developing countries. *Diagnostic Microbiology and Infectious Disease*. 2016; 84(1): 80-86. [doi:10.1016/j.diagmicrobio.2015.10.002](http://dx.doi.org/10.1016/j.diagmicrobio.2015.10.002)

Murray C et al. Global, regional, and national age-sex specific all-cause and cause-specific mortality for 240 causes of death, 1990-2013: a systematic analysis for the Global Burden of Disease Study 2013. *The Lancet*. 2015; 385(9963): 117-171. doi:10.1016/S0140-6736(14)61682-2.

Mushtaq F & Ritiche D. Do we know what people die of in the emergency department? *Emergency Medicine Journal*. 2015; 22: 718-721. doi: 10.1136/emj.2004.018721

Oluwasola OA, Fawole OI, Otegbayo AJ, Ogun GO, Adebamowo C a, Bamigboye AE. The autopsy: knowledge, attitude, and perceptions of doctors and relatives of the deceased. *Arch. Pathol. Lab. Med.* 2009;133(1):78-82. doi:10.1043/1543-2165-133.1.78.

Roulson J, Benbow EW, Hasleton PS. Discrepancies between clinical and autopsy diagnosis and the value of post mortem histology: a meta-analysis and review. *Histopathology*. 2005; 47(6): 551-9.

Sebire NJ, Weber MA, Thayyil S, Mushtaq I, Taylor A, Chitty LS. Minimally invasive perinatal autopsies using magnetic resonance imaging and endoscopic postmortem examination (“keyhole autopsy”): feasibility and initial experience. *The Journal of Maternal-Fetal & Neonatal Medicine.* 2012; 25(5):513-8. doi:10.3109/14767058.2011.601368.

Turner GDH, Bunthi C, Wonodi CB, et al. The role of postmortem studies in pneumonia etiology research. *Clinical Infectious Diseases.* 2012; 54 Suppl 2(Suppl 2):S165-71. doi:10.1093/cid/cir1062.

Ugiagbe EE, Osifo OD. Postmortem examinations on deceased neonates: a rarely utilized procedure in an African referral center. *Pediatric and Developmental Pathology*. 2012; 15:1–4

Vogel G. How Do You Count the Dead ? *Science (80-. ).* 2012;336 (June 2012):1372-1374.

Weustink AC, Hunink MGM, Dijke CF Van, Renken NS, Krestin GP, Oosterhuis JW. Minimally invasive autopsy: an alternative to conventional autopsy? *Radiology*. 2009;250(3):897-904

# 13. Appendices

## Appendix 1. Lab and Diagnostics Plan

## Appendix 2: Informed Consent Form

## Appendix 3: Site Specific Addendum
